# Non-destructive debridement and tuneable ion release via magnesium abrasion and electro-dissolution promote bone regeneration and osseointegration of infected implants

**DOI:** 10.1016/j.mtbio.2026.103183

**Published:** 2026-04-30

**Authors:** Zhixiang Nie, Yasheng Sun, Ke Li, Jukka P. Matinlinna, William M. Palin, Liam M. Grover, Zhen Zhang

**Affiliations:** a Department of Stomatology, Union Hospital, Tongji Medical College, Huazhong University of Science and Technology, Wuhan, 430022, China; b School of Stomatology, Tongji Medical College, Huazhong University of Science and Technology, Wuhan, 430030, China; c Hubei Province Key Laboratory of Oral and Maxillofacial Development and Regeneration, Wuhan, 430022, China; d State Key Laboratory of Materials Processing and Die & Mould Technology, School of Materials Science and Engineering, Huazhong University of Science and Technology, Wuhan, 430074, China; e Biomaterials Science, Division of Dentistry, School of Medical Sciences, The University of Manchester, M13 9PL, UK; f Dental Materials Science, Applied Oral Sciences, Faculty of Dentistry, The University of Hong Kong, Hong Kong SAR; g University of Birmingham, College of Medical and Dental Sciences, Institute of Clinical Sciences, 5 Mill Pool Way, Edgbaston, Birmingham, B5 7EG, UK; h Healthcare Technologies Institute, School of Chemical Engineering, University of Birmingham, Birmingham, UK

**Keywords:** Metallic implants infection, Magnesium abrasion, Electro-dissolution, Bone regeneration, Osseointegration

## Abstract

Infection of metallic implants remains a major clinical challenge, often leading to failure and the need for revision. One approach to the treatment of infection is by debridement to remove infected tissue and biofilm – unfortunately this damages the implant surface and compromises secondary osseointegration. We report a non-destructive, dual-function strategy: magnesium abrasion and electro-dissolution (MAE) that simultaneously achieves surface decontamination, osseo-regeneration, and secondary osseointegration. MAE utilizes, magnesium particles to mechanically disrupt biofilms since magnesium is softer than titanium, it does not damage the implant surface. Subsequent electro-dissolution removes excess magnesium to avoid prolonged exposure, maintaining a transient immune-stimulatory window. *In vivo*, MAE modulates early inflammation by promoting neutrophil apoptosis, reducing neutrophil extracellular traps (NETs) formation, and polarizing macrophages toward a reparative M2 phenotype. Transcriptomic analysis reveals downregulation of key inflammatory pathways, supporting an anti-inflammatory, pro-regenerative immune environment. These immune effects facilitate enhanced bone regeneration, including improved collagen deposition, trabecular organization, and accelerated mineralization of newly formed bone. Importantly, MAE-treated implants achieve robust secondary osseointegration. This study highlights the clinical potential of MAE as a surface-preserving strategy for managing infected metallic implants by integrating *in situ* debridement with immune modulation without requiring implant removal or invasive revision surgery.

## Graphical abstract

**Figure:**
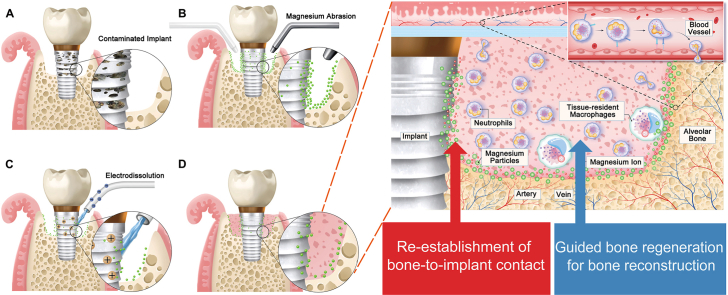

## 1 Introduction

Metals and metal alloys, including stainless steel, titanium and its alloys, cobalt-based alloys, and nickel-titanium (NiTi) shape memory alloys have been extensively used in clinical applications owing to their high mechanical strength, excellent wear and corrosion resistance, superior fatigue performance, and favorable biocompatibility [[1], [2], [3], [4], [5]]. These materials serve as fundamental components in orthopedic and dental procedures, including bone regeneration, structural support, and prosthodontic reconstruction. However, the surfaces of such implants are inherently prone to bacterial contamination during surgical procedures, particularly from the skin or mucosal tissues. Notably, most conventional implants lack intrinsic antibacterial properties, rendering them highly susceptible to infection [6]. Peri-implant infections can lead to severe inflammation, progressive bone loss, and ultimately implant failure [7]. These complications often necessitate revision surgeries, resulting in increased clinical risk, patient morbidity, psychological burden, and healthcare costs [8]. Therefore, there is an urgent need for non-invasive and effective strategies that can eliminate biofilms from contaminated implant surfaces while preserving the potential for successful secondary osseointegration [9]. One such strategy is the removal of biofilm using mechanical agitation when the implant is *in situ*.

Despite the availability of various debridement modalities, effective salvage of infected implants remains clinically challenging as current strategies inevitably involve trade-offs between decontamination efficacy and surface preservation. Conventional biofilm removal predominantly relies on aggressive mechanical approaches, such as titanium brushes or ultrasonic surgical tools, which can effectively eliminate implant surface contaminants but irreversibly disrupt the implant's micro- and nanotopography—features now widely recognized as critical for osteoblast adhesion and subsequent osseointegration [10]. Alternatively, local applications of antibiotics, disinfectants, or laser therapy can access microstructured surfaces and effectively kill bacteria without physically damaging the implant. However, these methods are often insufficient for completely removing extracellular polymeric substances (EPS) that form the structural backbone of hazardous biofilms and protect embedded pathogens [11,12]. Surface abrasion using high-velocity particle streams driven by compressed air has emerged as a potentially effective decontamination strategy. Yet, currently used materials such as calcium phosphate and glycine may leave surface residues that adversely affect subsequent bone regeneration and osseointegration [13]. Consequently, these methods leave clinicians with a dilemma: choosing between surface preservation with incomplete decontamination, or effective cleaning at the expense of the implant's regenerative potential [14,15]. Hence, there remains a significant unmet need for abrasive media that not only enable thorough biofilm removal but also actively support osteogenesis and bone–implant reintegration.

Magnesium (Mg) and its alloys have attracted increasing interest in implant-related research due to their unique mechanical and biological characteristics. Owing to their lower hardness relative to titanium, Mg particles can remove biofilm contaminants while minimizing damage to the implant surface [[16], [17], [18]]. Moreover, Mg is a biodegradable metal whose degradation products have been shown to promote osteogenesis and enhance secondary osseointegration [19]. In particular, magnesium ions (Mg^2+^) have been reported to promote a pro-osteogenic immune microenvironment through TRPM7-dependent signaling and NRF2-mediated redox homeostasis, accompanied by the suppression of NF-κB–driven inflammatory responses [20,21]. However, the biological effects of Mg^2+^ are highly dose- and time-dependent, excessive or prolonged exposure to Mg^2+^ can activate NF-κB signaling, disrupt immune homeostasis, and inhibit extracellular matrix mineralization, ultimately impairing bone healing. [22,23]. These limitations underscore a key challenge in current Mg-based strategies: how to harness the beneficial immunoregulatory and osteogenic effects of Mg while avoiding adverse responses caused by uncontrolled degradation [24].

Electrochemical dissolution represents a promising solution to these challenges. This technique allows for the selective removal of active metallic components from alloy surfaces under controlled environmental conditions, enabling precise regulation of surface composition and residual content [25,26]. Moreover, by applying electrical insulation to designated regions of the anode, the electro-dissolution process can be spatially and temporally controlled. When carefully regulated, this process can induce the early degradation of excess Mg^2+^ at the implant interface, maintaining magnesium levels within an optimal window to support the formation of a pro-regenerative immune niche during the critical early stages of healing [27,28], without impairing the bone healing process.

**Scheme 1:**
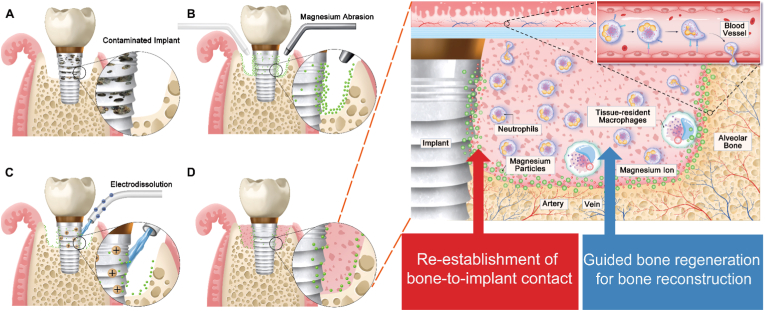
Schematic illustration of the MAE treatment process and its regulation of the local inflammatory microenvironment. **A)** Bacterial biofilm formation on the implant surface. **B)** Magnesium abrasion (MA) mechanically disrupts and removes bacterial colonization. **C)** Electro-dissolution controls the residual magnesium content on the implant surface. **D)** The MAE process maintains a bone-regeneration-friendly concentration of Mg, thereby facilitating re-osseointegration and promoting bone regeneration.

In this study, we present a novel, non-destructive surface treatment strategy that integrates magnesium abrasion and electro-dissolution (MAE) for the efficient removal of infectious layers from contaminated implant surfaces (Scheme 1). Unlike conventional mechanical or chemical approaches that either damage the implant or leave biofilm remnants, the MAE system achieves both effective implant decontamination, preservation of surface microstructure and functional integrity. We further hypothesize that the MAE strategy not only removes pathogens but also actively promotes peri-implant bone regeneration and secondary osseointegration, owing to the synergistic effects of: (1) magnesium abrasion, which gently disrupts and removes biofilms; and (2) electro-dissolution, which regulates residual Mg levels to modulate the local immune environment. Collectively, the development of the MAE system offers a clinically translatable and immunomodulatory approach for the treatment of implant-associated infections. It addresses the urgent need for non-invasive, surface-preserving biofilm removal while concurrently enhancing the biological potential for implant reintegration, thereby offering a path toward improved long-term outcomes in implant therapy.

## 2 Results

### 2.1 The decontamination of implant surface via MAE

**Fig. 1:**
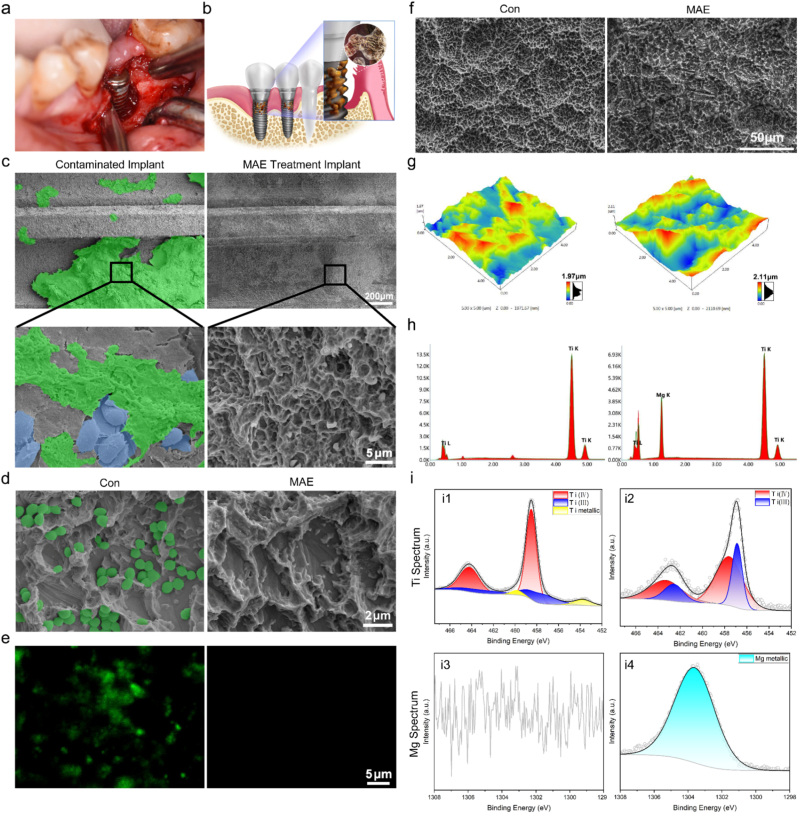
MAE-mediated biofilm removal and characterization of implants before and after treatment. **a)** Clinical observation showing significant bone loss around the implant following surgical debridement. **b)** Schematic illustration of peri-implantitis accompanied by alveolar bone loss. **c)** SEM images showing biofilm (green) and inflammatory cells (blue) on contaminated and MAE-treated dental implants. **d)** SEM image illustrating *Staphylococcus aureus* adhesion on the implant surface, which is effectively removed by MAE treatment. **e)** Live/dead fluorescence imaging of the implant surface before and after MAE treatment **f, g)** Comparison of surface nano- and micro-topography of untreated and MAE-treated implants via SEM and AFM imaging. **h)** EDS spectrum showing residual magnesium on the implant surface post-MAE treatment. **i)** XPS spectra of titanium and magnesium on implant surfaces before and after MAE treatment. (For interpretation of the references to colour in this figure legend, the reader is referred to the Web version of this article.)

Clinically, bone loss around the implant was observed after the mucosal flap was opened (Fig. 1a), and schematically illustrated in Fig. 1b. The contaminated implant, characterized by severe bone loss, was extracted and examined using SEM. Spherical Mg particles (Fig. S1, Supporting Information) were expelled from the spray gun to decontaminate the affected implant (Fig. S2, Supporting Information). The plaque biofilm on the implant surface was marked in green and removed using MAE (Fig. 1c).

*Staphylococcus aureus* (*S. aureus*) was initially employed as a representative pathogen to evaluate the decontamination efficacy of MAE on infected implant surfaces. Scanning electron microscopy (SEM) images revealed dense spherical bacteria adhering to untreated implant surfaces, whereas MAE treatment resulted in complete removal of surface-associated bacteria without apparent damage to the underlying microstructure (Fig. 1d). Consistently, live/dead fluorescence staining demonstrated robust bacterial viability on control surfaces, while almost all bacteria were eliminated following MAE treatment (Fig. 1e). To further evaluate the antibacterial robustness of MAE, its efficacy was extended to both Gram-positive (*S. aureus*) and Gram-negative (*Escherichia coli* (*E. coli*)) bacteria. Live/dead fluorescence staining revealed that bacteria on untreated implant surfaces remained predominantly viable, whereas MAE-treated surfaces exhibited extensive bacterial death, with essentially no detectable residual live bacteria. (Fig. S3a, Supporting Information). Consistently, turbidity analysis demonstrated a marked reduction in bacterial growth following MAE treatment, as evidenced by significantly lower OD_450_ values compared with untreated controls for both bacterial strains (Fig. S3b–c, Supporting Information). Quantitative CFU analysis further confirmed that MAE treatment dramatically reduced viable bacterial counts, achieving a >99.7% clearance rate for both *S. aureus* and *E. coli* (Fig. S3d–f, Supporting Information). Together, these results demonstrate that MAE provides broad-spectrum antibacterial efficacy against both Gram-positive and Gram-negative bacteria, supporting its potential utility for the decontamination of infected metallic implants.

SEM observations revealed that the MA-treated implant surface lacked the characteristic microstructural features observed on the original implants. However, these microstructures were largely restored following electro-dissolution (Fig. 1f, Fig. S4a (Supporting Information)). Consistently, atomic force microscopy (AFM) analysis showed that the ten-point average surface roughness (Rz) of the original, MA-treated, and MAE-treated implants was 1.97 μm, 1.11 μm, and 2.11 μm, respectively (Fig. 1g, Fig. S4b (Supporting Information)). Compared to the original implant surface, the micro- and nanotopography of the implant, crucial for its biological function in vivo, was well preserved after MAE treatment, implying that although the magnesium particles enabled removal of the biofilm, they did not damage the implant surface. Energy dispersive spectrometry (EDS) analysis of the implant surface showed the presence of Mg remaining after MAE treatment (Fig. 1h). The X-ray photoelectron spectroscopy (XPS) spectrum of Ti 2p in the control group revealed peaks at 458.5 eV and 464.2 eV corresponding to Ti^4+^, while additional peaks at 457.8 eV and 463.5 eV were attributed to Ti^3+^. Overlapping peaks at 453.7 eV and 459.8 eV were resolved and assigned to metallic titanium (indicated in blue). After MA treatment, only weak Ti signals were detected due to surface deposition of magnesium. The XPS spectrum of Mg displayed a peak at 1303.7 eV, corresponding to Mg 1s. Following MAE treatment, the complete Ti spectrum reappeared on the surface, along with detectable Mg signals, indicating partial retention of Mg (Fig. 1i, Fig. S5c–d (Supporting Information)).

### 2.2 Neutrophils infiltrate into blood clots after MAE treatment

**Fig. 2:**
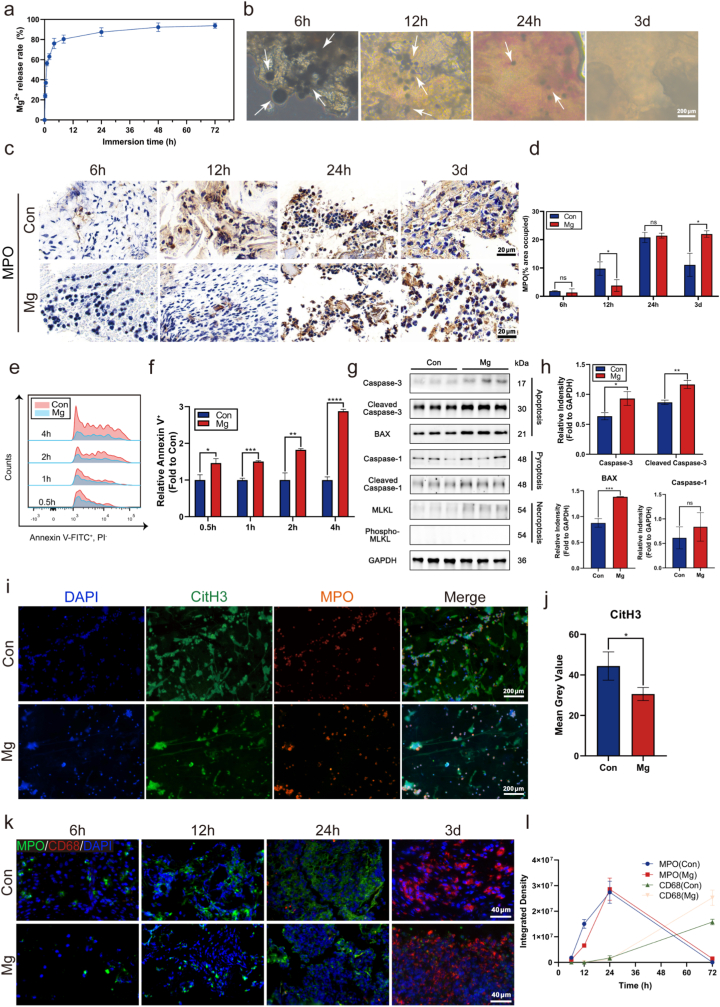
Magnesium particles modulate the subsequent inflammatory microenvironment by regulating early neutrophil responses. **a)***In vitro* degradation profile of magnesium particles in simulated body fluid (SBF) (n = 3). **b)***In vivo* bright-field microscopy images showing the morphology of degrading magnesium particles in alveolar bone. **c, d)** Immunohistochemical staining of myeloperoxidase (MPO) and quantification of MPO-positive areas in the blood clot at 6 h, 12 h, 24 h, and 3 days post-application of MAE-equivalent magnesium dose into rat first molar extraction sockets (n = 3). **e, f)** Flow cytometric analysis of neutrophil apoptosis following in vitro incubation with MAE-equivalent magnesium particles for 0.5 h, 1 h, 2 h, and 4 h (n = 3). **g, h)** Western blot analysis and semi-quantification of apoptosis-, pyroptosis-, and necroptosis-associated protein expression in neutrophils after 8 h of in vitro stimulation with MAE-equivalent magnesium particles (n = 3). **i, j)** Immunofluorescence imaging of neutrophil extracellular traps (NETs) formation after 8 h of in vitro stimulation with MAE-equivalent magnesium particles, showing co-localization of CitH3 (green), MPO (red), and DAPI (blue); quantitative analysis of mean gray value is provided (n = 3). k, l Immunofluorescence staining of blood clots from rat first molar extraction sockets for MPO (green), CD68 (red), and DAPI (blue) at 6 h, 12 h, 24 h, and 3 days post magnesium application, with quantification of total fluorescence intensity (n = 3). ns *P*> 0.05, ∗*P*< 0.05, ∗∗*P*< 0.01, ∗∗∗*P*< 0.001, ∗∗∗∗*P* < 0.0001 by Student's *t*-test. Error bars = SD; data are presented as mean values ± SD. (For interpretation of the references to colour in this figure legend, the reader is referred to the Web version of this article.)

Following MA treatment, the implant surface was covered with 1.08 ± 0.17 mg cm^−2^ of Mg particles (Fig. S5a, Supporting Information). Subsequently, the majority of these particles were removed during the MAE process, leaving approximately 20% residual magnesium on the surface (Fig. S5b, Supporting Information). Notably, this residual amount was associated with enhanced cellular activity (Fig. S5c, Supporting Information). Mg particles exhibited rapid degradation in simulated body fluid (SBF), with 87.4 wt% (±4.2 wt%) of the material degraded within 24 h and 93.8 wt% (±2.5 wt%)) by day 3 (Fig. 2a). *In vivo* results showed a consistent trend: distinct Mg particles were visible at 6 h post-implantation, gradually decreased in size and number over time, with only a few particles remaining at 24 h, and becoming nearly undetectable by day 3 (Fig. 2b), aligning well with the in vitro degradation profile. Hematoxylin-eosin (H&E) staining of the blood clot at the bone defect site at different time points revealed reduced inflammatory cell infiltration in the surgical area following magnesium particles application (Fig. S6, Supporting Information). We assessed neutrophil infiltration using myeloperoxidase (MPO) immunohistochemical (IHC) staining. From 6 to 24 h, neutrophil counts increased progressively, followed by a decline from 12 h to 3 days. Notably, neutrophil infiltration was significantly lower in the Mg-treated group compared to controls at the 12-h time point, but increased so that it was significantly higher at 3d (Fig. 2c and d). Flow cytometric analysis using Annexin V and propidium iodide (PI) double staining revealed a time-dependent increase in neutrophil apoptosis in the magnesium particle-treated group from 0.5 to 4 h, with apoptosis rates significantly higher than those in the control group (Fig. 2e and f). Immunofluorescence staining of apoptosis-related markers at 4 h further confirmed that co-culture with magnesium particles markedly promoted neutrophil apoptosis (Fig. S7, Supporting Information).

Western blot analysis of neutrophil apoptosis-, pyroptosis-, and necroptosis-related proteins showed that magnesium particle treatment significantly upregulated the expression of apoptotic markers, including Caspase-3, Cleaved Caspase-3, and BAX, compared to the control group. In contrast, pyroptosis-related proteins (Caspase-1, Cleaved Caspase-1) and necroptosis-associated proteins (MLKL, Phospho-MLKL) were minimally expressed or undetectable in both groups. These results suggest that magnesium particles promote neutrophil apoptosis rather than triggering inflammatory forms of cell death such as pyroptosis or necroptosis (Fig. 2g and h). Immunofluorescence (IF) co-localization of CitH3 (citrullinated histone marker) and MPO (neutrophil marker) revealed a reduction in both the number and spread area of NETs in neutrophils treated with magnesium particles. Semi-quantitative fluorescence analysis further supported this observation, confirming the suppressive effect of magnesium on NETs formation (Fig. 2i and j).

IF staining of the blood clot at the defect site for neutrophils (MPO) and macrophages (CD68) demonstrated a sequential pattern of cellular infiltration. Neutrophils peaked at the surgical site at 24 h post-injury, whereas only a limited number of macrophages were present at that time. By day 3, neutrophils were nearly absent in the defect area, while macrophages were abundantly distributed throughout the site (Fig. 2k and l).

### 2.3 Macrophages internalize Mg particles and shift toward an anti-inflammatory phenotype

**Fig. 3:**
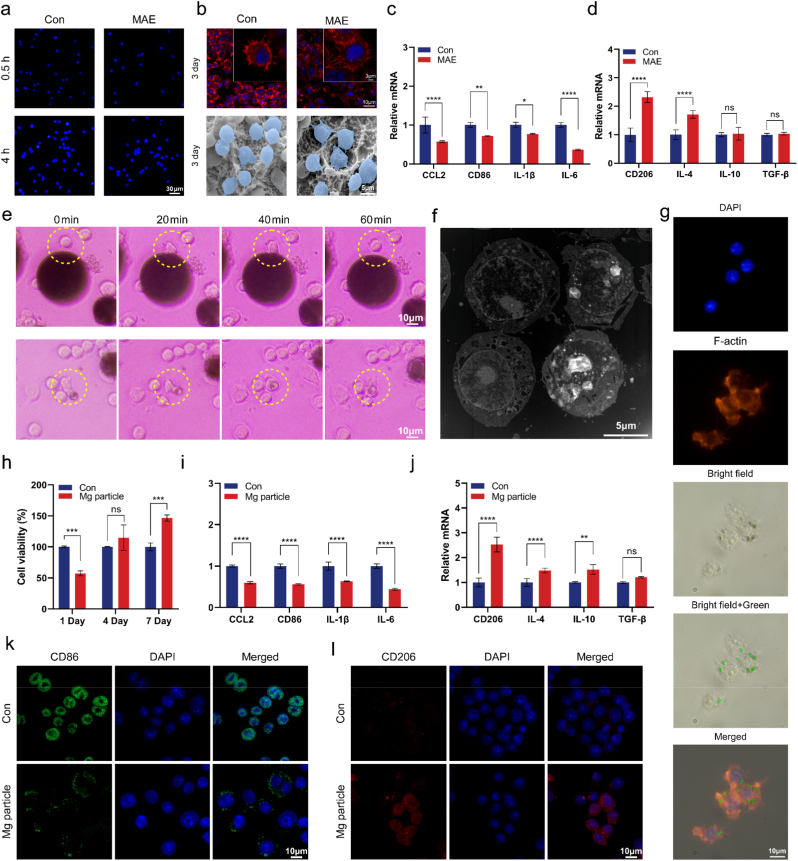
Effects of MAE-treated surfaces and Mg particles on macrophage behavior. **a)** Macrophages adhered on MAE-treated surface at 0.5 h and 4 h. **b)** Cell skeleton and morphology on the MAE treated surface after 3 days. **c, d)** Relative mRNA expression of inflammation-related genes. **e)** Bright-field microscopy showing macrophages engulfing small Mg particles (<5 μm) and transiently contacting larger particles (>10 μm). **f)** TEM confirming intracellular localization of phagocytosed Mg particles. **g)** Combining nuclear staining (blue), cytoskeleton staining (red) and Mg particles labeled with an alternative green colour, we observed that the Mg particles are indeed present intracellularly around the nucleus. **h)** CCK-8 assay showing macrophage proliferation at days 1, 4, and 7. **i, j)** Downregulation of pro-inflammatory genes and upregulation of anti-inflammatory genes on day 3 **k, l)** IF staining of CD86 (M1) and CD206 (M2) indicating polarization toward M2 phenotype. *n*= 3 per group. ns: not significant, ∗*P*< 0.05, ∗∗*P*< 0.01, ∗∗∗*P*< 0.001, ∗∗∗∗*P* < 0.0001 by Student's T-test. Error bars = SD; data are presented as mean values ± SD. (For interpretation of the references to colour in this figure legend, the reader is referred to the Web version of this article.)

Macrophages were observed to adhere to the MAE-treated surfaces as early as 0.5 and 4 h post-seeding (Fig. 3a). After 3 days of culture, confocal laser scanning microscopy (CLSM) images revealed that macrophages exhibited polygonal morphology with extended lamellipodia and filopodia on both control and MAE surfaces (Fig. 3b). Similar findings were confirmed via scanning electron microscopy (SEM), showing well-spread macrophages with abundant membrane protrusions on both surfaces. PCR showed that the mRNA levels of M1 macrophage markers—including CCL2, CD86, IL-1β, and IL-6—were significantly downregulated in the MAE group (Fig. 3c), while M2-associated markers CD206 and IL-4 were upregulated (Fig. 3d), suggesting a shift toward an anti-inflammatory phenotype. To investigate whether the anti-inflammatory response was related to phagocytosis of magnesium particles, macrophages were co-cultured with Mg particles of different sizes. It was observed that macrophages preferentially internalized smaller particles (<5 μm), while only transiently interacting with larger ones before disengaging (Fig. 3e). To further confirm particle internalization, both transmission electron microscopy (TEM) and fluorescence microscopy were employed, clearly demonstrating intracellular localization of smaller Mg particles within the cytoplasm (Fig. 3f and g).

Although a transient decrease in cell viability was observed initially in the Mg particle group, macrophages exhibited a robust proliferation response over time (Fig. 3h). mRNA levels of pro-inflammatory cytokines (CCL2, CD86, IL-1β, and IL-6) were significantly reduced, whereas anti-inflammatory and tissue repair-associated genes (CD206, IL-4, and IL-10) were markedly upregulated (Fig. 3i and j). IF staining further supported this phenotype switch, showing reduced surface expression of the M1 marker CD86 and increased expression of the M2 marker CD206 in the Mg particle group (Fig. 3k and l).

### 2.4 Transcriptome sequencing gene analysis of macrophage

**Fig. 4:**
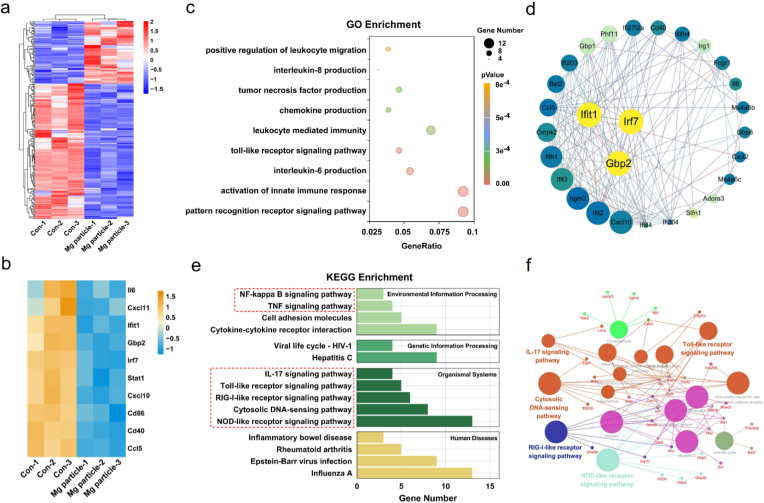
RNA sequence analysis of RAW264.7 co-cultured with Mg particles. **a)** Heatmap of the distinct upregulated and downregulated macrophage genes after coculture with Mg particles. **b)** Heatmap of the distinct inflammation-related genes of Mg particle vs Con. **c)** GO pathway enrichment analysis of Mg particle vs Con. **d)** Protein interactions analysis of Mg particle vs Con. **e)** Bagplot of KEGG enrichment between the Ti and Mg particle groups. **f)** KEGG pathway interaction analysis of immune-related genes within Mg particle vs Con.

Transcriptomic analysis further elucidated the molecular mechanisms by which Mg particles activate macrophages and induce phenotypic switching toward an anti-inflammatory state (Fig. 4a). Deep RNA sequencing confirmed widespread downregulation of key pro-inflammatory genes in the Mg particle group compared to the control (Fig. 4b). Gene Ontology (GO) enrichment analysis revealed that downregulated genes in the Mg-treated group were predominantly associated with inflammation-related pathways, including pattern recognition receptor signalling, innate immune activation, interleukin-6 production, Toll-like receptor signalling, and tumor necrosis factor (TNF) production (Fig. 4c). Protein–protein interaction (PPI) clustering of differentially expressed genes revealed that the downregulated genes were closely linked to inflammatory biological functions, further supporting the immunomodulatory effect of Mg particles (Fig. 4d). Kyoto Encyclopedia of Genes and Genomes (KEGG) pathway enrichment analysis identified significantly affected signalling pathways, highlighting the role of Mg particles in promoting macrophage polarization toward the M2 phenotype by inhibiting inflammation-associated transcriptional programs (Fig. 4e). Network analysis of immune-related proteins and signalling pathways revealed that the downregulated proteins were primarily associated with NOD-like receptor signaling, RIG-I-like receptor signalling, cytosolic DNA-sensing pathways, IL-17 signalling, and Toll-like receptor signalling (Fig. 4f). Taken together, these findings indicate that Mg particles suppress pro-inflammatory signaling cascades in macrophages, leading to downregulation of inflammatory gene expression. This immunoregulatory effect would be likely prevent failure and fibrosis of the implant, thereby enabling superior osseointegration.

### 2.5 MAE treatment enhances early bone regeneration

**Fig. 5:**
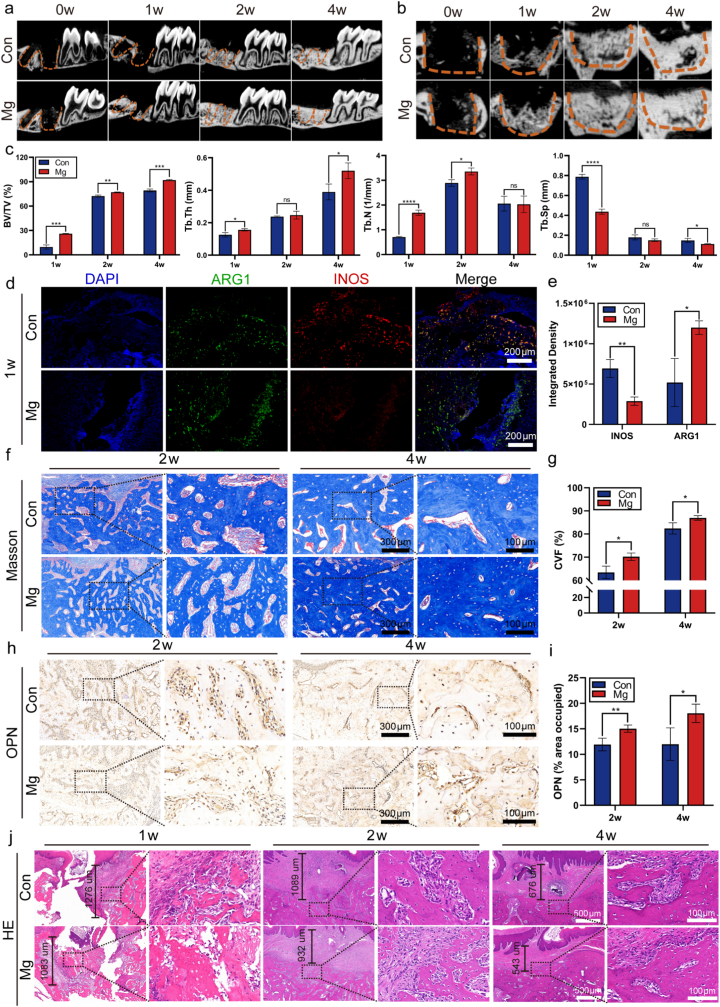
Magnesium particles promote bone regeneration in rat maxillary bone defects. **a, b)** Representative sagittal (a) and coronal (b) micro-CT images of rat maxillary bone defects at indicated time points following implantation of MAE-equivalent doses of magnesium particles. **c)** Quantitative analysis of bone regeneration based on BV/TV (bone volume/total volume), Tb. Th (trabecular thickness), Tb. N (trabecular number), and Tb. Sp (trabecular separation) in rat femoral defects (n = 3). **d, e)** Immunofluorescence staining of iNOS (red) and ARG1 (green) at 1 week post-implantation reveals localized pro- and anti-inflammatory macrophage expression at the defect site (n = 3). **f, g)** Masson's trichrome staining of coronal sections shows increased collagen deposition in defects treated with MAE-equivalent magnesium particles (n = 4); high-magnification views of boxed regions are shown on the right. **h, i)** Representative immunohistochemical staining of OPN and corresponding semi-quantitative analysis (n = 4); boxed regions are enlarged on the right. **j)** H&E staining at 1, 2, and 4 weeks post-implantation reveals reduced vertical distances from the highest point of newly formed bone to the lowest point of epithelium in magnesium-treated groups (n = 4). ns *P*> 0.05, ∗*P*< 0.05, ∗∗*P*< 0.01, ∗∗∗*P*< 0.001, ∗∗∗∗*P* < 0.0001 by Student's *t*-test. Error bars = SD; data are presented as mean values ± SD. (For interpretation of the references to colour in this figure legend, the reader is referred to the Web version of this article.)

Mg particles promoted the proliferation and osteogenic differentiation of BMSCs in vitro (Fig. S9 and S10, Supporting Information). Sagittal and coronal micro-CT images demonstrated progressive healing of the extraction sockets from week 0 to week 4. Notably, the sockets treated with magnesium particles exhibited denser new bone formation compared to those without Mg treatment (Fig. 5a and b). Quantitative analysis showed that bone volume fraction (BV/TV) increased steadily from week 1 to week 4, with significantly higher values in the MAE group at all time points. Similarly, trabecular thickness (Tb. Th) increased over time and was consistently greater in the Mg-treated group than in controls. Trabecular number (Tb. N) rose from week 1 to week 2 and was higher in the MAE group; however, a decrease was observed by week 4 due to increased mineralization. Correspondingly, trabecular separation (Tb. Sp) declined from week 1 to week 4, with significantly lower values in the MAE group at both week 1 and week 4 (Fig. 5c).

IF staining for iNOS (M1 marker) and Arg1 (M2 marker) was performed at week 1. The results showed reduced iNOS and increased Arg1 expression in the Mg-treated group compared to the control, indicating that magnesium promotes an anti-inflammatory, pro-regenerative macrophage phenotype during the early healing phase (Fig. 5d).

Histological analysis using Masson's trichrome staining revealed greater collagen deposition in the magnesium-treated group compared to the control group (Fig. 5e and f). IHC staining of the osteogenic marker collagen type I (COL-1) further supported these findings (Fig. S11, Supporting Information). The expression level of osteopontin (OPN), an osteogenic marker, was used to localize and identify regions of active bone formation. IHC analysis confirmed that OPN expression was higher in the magnesium-treated group compared to the control group (Fig. 5h–i). H&E staining of coronal sections at weeks 1, 2, and 4 showed more prominent new bone trabeculae in the magnesium group. Quantification of the vertical distance from the nadir of the osseous defect to the stratum spinosum demonstrated accelerated bone regeneration in the magnesium group when compared with the control (Fig. 5j, Fig. S12 (Supporting Information)). Representative H&E staining of the heart, liver, spleen, lungs, and kidneys at 4 weeks post-implantation revealed no apparent solid lesions or histopathological abnormalities in either group, indicating favorable biocompatibility and systemic safety of the implanted magnesium particles (Fig. S13, Supporting Information).

### 2.6 Re-osseointegration of MAE-treated implants surface

**Fig. 6:**
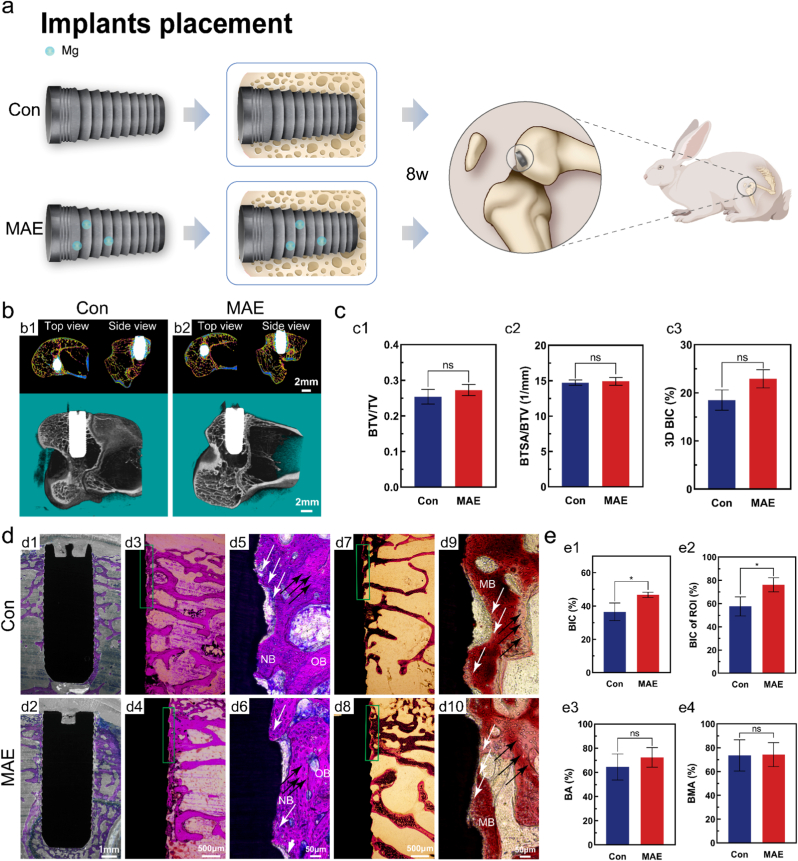
*In vivo* evaluation of the osseointegration ability of MAE-treated implants. **a)** Schematic illustration of the rabbit model used for in vivo experiments. **b)** Micro-CT images of the implants and the peri-implant bone tissue. **c)** Quantitative analysis of the bone tissue volume/total volume (BTV/TV), bone surface area/total bone volume (BSTA/BTV), and 3D bone-implant contact (3D BIC) obtained from the micro-CT. **d)** Histological staining of peri-implant bone tissue by methylene blue acid fuchsin and Alizarin Red. **e)** Analysis of the bone-implant contact (BIC), BIC in the region of interest (BIC of ROI), bone area (BA), and bone mineralization area (BMA). Abbreviations: NB, new bone; OB, old bone; MB, mineralized bone; black arrow, osteoblasts; white arrow, osteocytes. *n* = 3 animals per group. *∗P* < 0.05, two-tailed *t*-test. *ns*, not significant. (For interpretation of the references to colour in this figure legend, the reader is referred to the Web version of this article.)

A New Zealand rabbit model was used to investigate the osseointegration effect of MAE-treated implants in vivo. Implants from the control and MAE-treated groups were surgically inserted into the distal femurs of rabbits and analyzed after eight weeks (Fig. 6a).

Micro-CT-based morphological analyses after eight weeks showed dense cortical bone around the MAE-treated implants without any signs of infection or inflammation (Fig. 6b). Statistical analyses of the region of interest indicated comparable results for bone tissue volume/total volume (BTV/TV) and bone surface area/total bone volume (BSTA/BTV), with improved 3D bone-implant contact (3D BIC) in the MAE-treated group (Fig. 6c1-c3). The direct structural connection between living bone and implant surfaces indicated successful osseointegration (Fig. 6d1-d6). Osteocytes and osteoblasts were distributed in the bone tissue surrounding the implants, with increased new bone formation in the MAE-treated group. Alizarin Red staining demonstrated bone tissue connections across all implant surfaces, indicating mineralized bone formation (Fig. 6d7-d10). Histological sections of the region of interest revealed a higher bone–implant contact (BIC) in the coronal region and cortical bone of the MAE group, suggesting enhanced osseointegration and bone regeneration at the interface (Fig. 6e1, e2). While quantitative analysis of bone area (BA) and bone mineralization area (BMA) showed no statistically significant differences between groups (Fig. 6e3, e4), the overall results confirmed successful osseointegration in the rabbit model, with superior outcomes in the MAE-treated group.

## 3 Discussion

In this study, we propose a dual-functional surface treatment strategy combining magnesium abrasion and electro-dissolution (MAE), which fundamentally differs from existing debridement technologies in both mechanistic basis and biological consequences. Conventional mechanical debridement approaches, including titanium brushes and ultrasonic devices, primarily rely on high mechanical forces to disrupt biofilms, inevitably causing irreversible damage to the micro- and nanotopographical features of implant surfaces that are essential for osteoblast attachment and secondary osseointegration [18]. In contrast, MAE employs biodegradable magnesium particles with lower hardness than titanium, enabling effective biofilm disruption while largely preserving the original surface architecture. This decoupling of decontamination efficacy from surface damage addresses a long-standing clinical dilemma that remains unresolved by current protocols. (Table S1, Supporting Information) [29,30]. Importantly, MAE is compatible with existing air-abrasion and electrochemical control systems, suggesting that its implementation does not require fundamentally new clinical infrastructure. Together with the scalable manufacturability of magnesium microparticles using established industrial processes, this feature supports the feasibility of MAE as a clinically translatable and potentially cost-effective implant salvage strategy.

Beyond its physical debridement advantage, MAE introduces a distinct mechanistic paradigm by integrating controlled magnesium electro-dissolution as an active immunomodulatory cue. Unlike passive surface-preserving or bactericidal strategies, MAE creates a controllable micro-environment for Mg^2+^ that actively shapes early immune responses, promoting neutrophil apoptosis over inflammatory cell death and biasing macrophage polarization toward a reparative phenotype [31,32]. This temporally regulated immune modulation establishes a pro-regenerative microenvironment that supports extracellular matrix remodeling and osteogenic signaling. In this context, magnesium degradation is not merely a material byproduct, but a temporally regulated biological trigger that couples surface decontamination with immune-guided bone regeneration.

The attachment and proliferation of macrophages and bone marrow mesenchymal stem cells (BMSCs) were not observed on the MA-treated surface (Fig. S8, Supporting Information). This was likely due to the presence of a substantial amount of residual Mg particles, which can generate hydrogen gas and create an alkaline microenvironment during degradation—conditions known to impair cellular adhesion and viability [20]. The early immune microenvironment, particularly within the first 7 days post-implantation, plays a pivotal role in determining the outcome of subsequent tissue regeneration [33,34]. After MAE treatment, residual magnesium on the implant surface undergoes gradual degradation, releasing Mg^2+^ that actively modulate innate immune activity. The presence of both magnesium particles and their degradation products create a favorable temporal window to guide host immune responses, which appears central to the therapeutic efficacy of MAE [35,36].

During the initial immune phase, neutrophils exhibited a delayed infiltration pattern post-MAE, aligning with previous findings that Mg^2+^ can enhance endothelial barrier integrity and restrict leukocyte transmigration by tightening intercellular junctions [37]. This likely contributes to dampening excessive acute inflammation. Upon activation, neutrophils may undergo apoptosis, pyroptosis, or necroptosis. Unlike the pro-inflammatory nature of pyroptosis and necroptosis, which involve the uncontrolled release of intracellular contents, apoptosis is a programmed form of cell death that preserves membrane integrity and facilitates resolution of inflammation through macrophage efferocytosis [38,39]. Mechanistically, BAX initiates the mitochondrial apoptosis pathway, leading to activation of Cleaved Caspase-3, which executes apoptosis by cleaving substrates such as PARP, Lamin, and DFF45. In contrast, Cleaved Caspase-1 and Phospho-MLKL are signature markers of pyroptosis and necroptosis, respectively [40,41]. Rather than broadly suppressing neutrophil recruitment, MAE selectively biases early neutrophil fate toward apoptosis while avoiding inflammatory cell death pathways. This pattern is distinct from conventional antimicrobial strategies, which often exacerbate NET-associated tissue damage, and suggests that magnesium acts as an immune ‘resolution cue’ rather than a simple anti-inflammatory agent [[42], [43], [44], [45]].

As the inflammatory cascade progresses, macrophages gradually emerge as the dominant immune population at the implantation site. While prolonged exposure to magnesium has been shown to impair osteogenesis, macrophages contribute to immune homeostasis by clearing residual submicron-to-micron-sized Mg particles through phagocytosis [23,46,47]. Notably, Zhang et al. demonstrated that macrophages can retain their viability, membrane integrity, and immunological function following the internalization of Mg particles [[48], [49], [50]]. Previous studies have reported immunomodulatory effects of Mg^2+^, yet often under conditions of sustained exposure that impair osteogenesis. In contrast, the MAE system establishes a transient Mg signaling window, enabling macrophage polarization toward a reparative phenotype without prolonged inflammatory stress. This temporal control may explain the favorable immune–regenerative coupling observed here [51,52] Transcriptomic and pathway analyses revealed broad suppression of well-established pro-inflammatory signaling cascades following MAE treatment. Importantly, beyond the downregulation of individual genes, pathway-level analysis indicated a systemic attenuation of core inflammatory networks, suggesting an active resolution of inflammation and mitigation of tissue damage [53]. These findings highlight that macrophage responses to magnesium extend beyond debris clearance: by adopting a reparative phenotype, they help shape a regenerative immune microenvironment that supports subsequent osteogenesis.

Consistent with this mechanism, MAE induced coordinated regulation of neutrophil and macrophage activity, resulting in improved bone regeneration outcomes. Rather than relying solely on aggressive antimicrobial clearance, these findings highlight the importance of early immune resolution in enabling successful secondary osseointegration [[54], [55], [56]]. Compared to conventional abrasive materials, the MAE approach yielded superior osteogenic outcomes across all time points, as reflected by more organized bone architecture and improved tissue maturation [[57], [58], [59]].

Notably, these biological effects led to successful secondary osseointegration, with regenerated bone forming intimate contact with the MAE-treated implant surface [60,61]. This integration highlights the translational potential of MAE as a surface engineering strategy that simultaneously addresses infection control and immune-guided bone healing. Importantly, the therapeutic efficacy of MAE relies on the temporally controlled release of Mg^2+^, which can in principle be regulated through adjustment of electro-dissolution parameters (e.g., current intensity and duration), as well as particle size and local microenvironmental conditions. Nevertheless, achieving precise and patient-specific control over magnesium degradation kinetics remains a key challenge for clinical implementation. By combining controlled material degradation with targeted immunomodulation, MAE provides a promising framework for developing next-generation implant therapies.

Despite the encouraging immunomodulatory and regenerative effects of MAE, several important questions remain. First, although MAE demonstrates robust antibacterial efficacy in vitro and effectively disrupts monospecies biofilms, direct in vivo evidence confirming bacterial clearance after treatment is still lacking. Given that implant-associated infections represent complex in vivo environments, this limitation should be acknowledged, and future studies incorporating relevant animal infection models—such as The New Zealand rabbit or Beagle peri-implantitis model—will be essential to validate the actual bacterial eradication capability of MAE under physiological conditions. Second, while MAE demonstrated efficacy against monospecies biofilms, clinical implant-associated infections often involve polymicrobial communities with enhanced biofilm resilience and immune evasion. Future studies should therefore evaluate MAE in multispecies biofilm models that better recapitulate clinically relevant infectious environments. Third, the current observation window captures the critical early phase of inflammation resolution and bone regeneration; however, it does not address long-term implant stability, bone remodeling dynamics, or performance under functional loading. Longitudinal studies incorporating extended follow-up and biomechanical assessments will be required to validate the durability of MAE-mediated implant salvage, particularly in chronic or severe infection settings. Beyond these temporal considerations, clinical implant infections also vary substantially depending on anatomical site and disease severity, such as localized peri-implantitis in periodontitis versus diffuse bone infection in osteomyelitis. These conditions differ in vascularization, immune cell infiltration, and bacterial burden, which may influence both magnesium degradation behavior and immune modulation. Establishing site-specific and severity-graded infection models will be important to more accurately define the therapeutic window and clinical indications of MAE.

From a translational perspective, parameters including spraying distance, particle flux, and electro-dissolution duration will require systematic optimization across different implant geometries and surface designs, with careful evaluation of safety margins prior to clinical application. While the present study focuses on titanium-based implants, the underlying principle of magnesium abrasion followed by electro-dissolution is not intrinsically material-specific. Given the lower hardness of magnesium relative to commonly used implant alloys, such as cobalt–chromium and stainless steel, MAE may offer a similarly surface-preserving debridement strategy for these materials. Nonetheless, dedicated validation on dissimilar metallic substrates is necessary to confirm efficacy and safety. Addressing these limitations will be essential to advance MAE from proof-of-concept toward robust clinical translation.

## 4 Conclusion

In summary, we have developed a non-destructive, dual-functional strategy—magnesium abrasion and electro-dissolution (MAE)—for *in situ* decontamination and immunomodulation of infected metallic implants. MAE efficiently removes bacterial biofilms without compromising the implant's surface topography, while the selectively retained magnesium transiently modulates the peri-implant immune response. This immunological fine-tuning, characterized by reduced neutrophil-driven inflammation and enhanced M2 macrophage polarization, fosters a regenerative microenvironment conducive to bone healing and secondary osseointegration. Importantly, the degradable magnesium layer can be precisely regulated via electrical control, offering a unique advantage over conventional mechanical or chemical methods. Taken together, our findings highlight the clinical potential of MAE as a surface-preserving, immune-responsive intervention for managing infected implants, bridging antimicrobial therapy and regenerative medicine without the need for implant removal.

## 5 Experimental section

### 5.1 Materials and reagents

Anti-MPO (Abcam, ab208670, UK), Anti-CD68 (Boster, BA3638, China), Anti-ARG1 (Proteintech, 16001-1-AP, USA), Anti-iNOS (Proteintech, 18985-1-AP, USA), Anti-OPN (Servicebio, GB11500, China), Anti-COL1 (Abcam, ab270993, UK), Anti-BAX (ABclonal, A19684, China), Anti-cleaved caspase-3 (ABclonal, A19664, China), Anti-caspase-3 (ABclonal, A11319, China), Anti-cleaved caspase-1 (ABclonal, A18646, China), Anti-caspase-1 (ABclonal, A16792, China), Anti-MLKL (ABclonal, A5579, China), Anti-phospho-MLKL (ABclonal, AP1174, China), Anti-GAPDH (ABclonal, A19056, China), FBS Premium (PAN-Biotech, ST30-3302, Germany), Poly-L-lysine (Beyotime, ST508, China).

### 5.2 Sample preparation and MAE treatment

In vitro experiments were conducted utilizing pure Ti plates (Grade 4, 99.6% purity, 14 mm diameter, 1 mm thickness). In contrast, pure Ti implants (Grade 4, 99.6% purity, 8 mm length, 3 mm diameter) were employed in vivo (Baoji Titanium Industry Co., Ltd.). The magnesium particles used in this study consisted of spherical metallic Mg particles produced by a gas atomization process under an inert atmosphere, which minimizes surface oxidation and ensures high purity. The powder was supplied by Tangshan Weihao Magnesium Powder Co., Ltd. (Tangshan, China). According to our characterization, the Mg particles exhibited a metallic state with a smooth surface morphology and a micron-scale particle size distribution, suitable for controlled abrasion without damaging the implant surface. The Ti specimens were sandblasted with 0.25–0.50 mm corundum grit at 5 bar for 1 min. Subsequently, the implants were acid-etched in hydrochloric acid/sulfuric acid (1:1) at 65 °C for 30 min. After the above treatments, the implants were ultrasonically cleaned for 15 min in acetone, ethanol (70%) and deionized water, and finally dried at room temperature. The control group comprised samples that had undergone sandblasting, large-grain sand treatment, and acid etching (SLA). To facilitate Mg particle spraying, the head of the sandblasting apparatus (SINOL, China) was positioned at a distance of 4-5 mm from the specimen surface, and operated with a pressure of 550 KPa. The samples from the control group were subjected to magnesium abrasion (MA) and subsequently classified as the MA group. MA samples were treated using a prototype electrolytic oxidation device operated at 15 V, using a flow rate of 800 rpm, at 2 mm distance from the sample surface, and an oxidation time of 20 s. The samples treated with magnesium abrasion and electro-dissolution (MAE) were classified as the MAE group.

### 5.3 Material surface analysis

Scanning electron microscopy (SEM; Tescan Escan, Clara, Czechia) was employed to observe the surface morphology, and the elemental composition of the implants were detected by an energy-dispersive X-ray spectrometer (EDS; Tescan Escan, Clara, Czechia) integrated into the SEM equipment. Surface topography was characterized using a large-sample atomic force microscope (Nanoscope Ⅳ, Nanoscope Systems, Korea) operated in tapping mode under ambient conditions. The chemical compositions of the implant surfaces were detected using X-ray photoelectron spectroscopy (XPS; AXIS Supra+, Shimadzu Co., Japan). The binding energies were calibrated using the C 1s (hydrocarbon C−C, C−H) of 284.6 eV, and the chemical states of Ti and Mg were analyzed.

### 5.4 Bacterial test procedures

*Staphylococcus aureus* (ATCC 6538) and *Escherichia coli* (ATCC 25922) were respectively used as representative Gram-positive and Gram-negative bacteria to evaluate the antibacterial efficacy of the MAE treatment, based on previously established protocol [24,62]. Briefly, bacteria was inoculated(≈10^6^ CFU, 1 ml) in an LB medium (Solarbio, L1010) at 37 °C for 12 h to obtain mid -logarithmic growth. Following a 12 h co-culture period, the implant surface was subjected to MAE treatment for bacterial removal. The samples containing bacteria were washed with PBS, fixed with glutaraldehyde (2.5%) for 4 h at 4 °C, and subsequently dehydrated in ethanol. Following sputter-coating with gold, the morphology of the bacteria was observed using SEM. The Live/Dead Baclight Bacterial Viability Kit (MKBio, Shanghai, MX4234) was used to quantify the viability of adherent bacteria on each sample in accordance with the manufacturer's instructions. The fluorescence images were taken on a confocal microscope and collected by NIS software (Nikon A1-Si, Japan). For turbidity analysis, the samples after MAE treatment were further cultured for another 12 h, bacterial suspensions collected after incubation were transferred to 96-well plates, and optical density at 450 nm (OD_450_) was measured using a microplate reader. For colony counting, bacterial suspensions were serially diluted (1 × 10^5^ and 1 × 10^6^), plated onto LB agar plates, and incubated at 37 °C for 24 h. Colony-forming units (CFU) were counted, and bacterial clearance rates were calculated using the following formula:

$$\text{Clearance}\;\text{rate}\;\left(\%\right)=\left(1-\frac{{\text{CFU}}_{\text{MAE}}}{{\text{CFU}}_{\text{Con}}}\right)\times 100\%\text{.}$$

### 5.5 Mg particles immersion experiment and Mg^2+^ quantification

MA treatment was applied to the top surface of 14 mm-diameter titanium discs. DMEM medium containing FBS (10%) was used to mimic the in vivo environment. 10 mL of FBS solution was poured into a sterile Petri dish, and the samples were soaked at 37 °C and 5% CO_2_. Fluid loss due to evaporation was replenished in a timely manner (∼300 to 400 μL every 24 h). Samples were taken at 0.25, 0.5, 1, 2, 4, 8, 24, 48, 96, and 144 h, respectively, and the Mg^2+^ concentration was measured according to the manufacturer's instructions of the Mg^2+^ detection kit (Solarbio, BC8337). The amount of Mg^2+^ dissolved at time i was given by Equation (1):

(1) Ci=(Ai/A0)×0.823

where Ci was the concentration (mM) of Mg^2+^ collected at time i. At time i, the dissolution rate of Mg^2+^ per unit surface area (mM cm^−2^) Ri was obtained from Equation (2):

(2) Ri=Ci/S0

where S0 was the top surface area (cm^2^) of the titanium disc.

### 5.6 Neutrophil dynamics in bone defect sites

Male Sprague-Dawley (SD) rats (100–150 g, Hunan SJA Laboratory Animal Co., Ltd) underwent extraction of the maxillary first molar under anesthesia. In the Mg group, 1 mg of magnesium particles was placed into the alveolar socket immediately after tooth extraction, while no intervention was performed in the control group. At 6 h, 12 h, 24 h, and 3 d post-surgery, blood clot tissues at the defect site were harvested. The degradation state of Mg particles was observed using an optical microscope (Nexcope NIB600, China), and images were recorded. Tissues were fixed in paraformaldehyde (PFA, 4%), embedded in paraffin, and sectioned at a thickness of 4 μm. Hematoxylin and eosin (H&E) staining was used for histological analysis. Immunohistochemistry for myeloperoxidase (MPO) was performed to detect neutrophil infiltration. The immunohistochemistry is supported by Wuhan PinuofeiBiological Technology Co., Ltd. Immunofluorescence staining for CD68 and MPO was also carried out. Fluorescence images were acquired using a Nikon A1 confocal microscope and analyzed with ImageJ software.

### 5.7 Mechanistic evaluation of Mg-induced neutrophil inflammation resolution

Bone marrow was harvested from the femurs and tibiae of male SD rats (100–150 g) after euthanasia with an overdose of pentobarbital sodium (100 mg/kg). Neutrophils were isolated using a Rat Bone Marrow Neutrophil Isolation Kit (Solarbio, P2610). Cells were seeded into 24-well plates and co-cultured with Mg particles (0.2 mg/mm^2^, equivalent to the residual dose in the MAE-treated surface). The control group received no treatment.

Neutrophil apoptosis was assessed at 0.5, 1, 2, and 4 h by flow cytometry using FITC-Annexin V/PI double staining (Beyotime, C1062S). Cells were blocked in BSA (1%), incubated at 4 °C for 30 min in the dark, washed with PBS, centrifuged at 800 rpm, and resuspended in PBS for analysis on a FACSCalibur flow cytometer (BD Biosciences, USA), followed by analysis in FlowJo v10. At 4 h, cells were also fixed for immunofluorescence and observed under a Nikon A1 confocal microscope. For protein analysis, cells were lysed in RIPA buffer containing protease inhibitors, and total protein was quantified using a BCA assay (Beyotime, P0012S). Western blot was performed using SDS-PAGE and PVDF membranes. Membranes were blocked with Rapid Blocking Buffer (ABclonal, RM02956) and incubated with primary antibodies against BAX, cleaved caspase-3, caspase-3, cleaved caspase-1, caspase-1, MLKL, phospho-MLKL, and GAPDH, followed by HRP-conjugated secondary antibodies. Bands were visualized using a gel imaging system and analyzed in ImageJ.

### 5.8 Immunofluorescence detection of NETs

To evaluate NET formation, 14 mm glass coverslips were pre-coated with 0.1 mg/mL poly-L-lysine to promote neutrophil adhesion. Neutrophils were co-cultured with Mg particles for 4 h, then stimulated with 0.2 μg mL^−1^ phorbol 12-myristate 13-acetate (PMA, MedChemExpress, HY-18739) for an additional 4 h. Cells were fixed in 4% PFA, permeabilized with Triton X-100 (0.5%), and blocked with BSA (5%). Primary antibodies against MPO (Proteintech, 66177-1-Ig, 1:200) and citrullinated histone H3 (Abclonal, A22348, 1:200) were applied and incubated overnight at 4 °C. After washing, cells were incubated with Cy3-labeled anti-rat IgG and AF488-labeled anti-rabbit IgG (Beyotime, both 1:200) for 1 h at room temperature. Nuclei were counterstained with DAPI (1 μg/mL, Beyotime). Confocal images were captured with a Nikon A1-Si microscope and analyzed for mean gray value using ImageJ.

### 5.9 Cell experiment procedures

#### 5.9.1 Cell morphology

The cells on the samples were washed with PBS, fixed with glutaraldehyde (2.5%) for 4 h at 4 °C, and then dehydrated in ethanol. After being sputter-coated with gold, the morphology change of the cell was observed by SEM. To observe the cell adhesive behavior of macrophages after coculturing for 3 days with the samples from different groups, F-actin staining was performed by the following procedure: cells were first fixed with PFA (4%) for 20 min and permeated with Triton X-100 (0.1%) for 20 min. Subsequently, cells were blocked with BSA (5%) for 1 h, followed by incubation with TRITC-phalloidin (Solarbio, CA1610) for 25 min to label F-actin. Nuclear counterstaining was performed using DAPI for 7 min. Images were acquired using the Nikon A1-Si microscope.

#### 5.9.2 Biocompatibility evaluation

Biocompatibility was determined on cells using Cell Counting Kit-8 (CCK-8, Beyotime, C0038), and according to the manufacturers instructions. Briefly, cells were seeded into 48-well plates and cocultured with different specimens for 1, 4, and 7 days, respectively, to perform the CCK-8 assay.

#### 5.9.3 Real-Time quantitative polymerase chain reaction (PCR)

In 24-well plates, cells were co-cultured with the sample. BMSCs were cultured overnight and induced by osteogenic medium for seven days, whilst macrophages were cultured overnight and induced by lipopolysaccharide (LPS, Solarbio, RPM0001) for two days. Total RNA was extracted from the samples using TRIzol reagent (ABclonal, RK30129) and subsequently reverse-transcribed into cDNA using the HiScript IV RT All-in-One SuperMix for qPCR (+dsDNase) (HYCEZMBIO, HYR801) according to the manufacturer's instructions. Quantitative PCR was performed using the Universal SYBR qPCR Premix HyTaq TM II (HYCEZMBIO, HYQ506) on an ABI 7300 Real-Time PCR System (Applied Biosystems, USA). GAPDH was assigned as an internal reference for normalizing mRNA expression levels. Primer sequences for the target genes are provided in Table S1. The relative expression of each gene was analyzed using the 2^−ΔΔCt^ method.

#### 5.9.4 Immunofluorescence staining

After incubation with specimens as described previously, Macrophages were washed with PBS three times and fixed with PFA (4%) for 20 min. Subsequently, they were blocked with BSA (5%) for 1 h, followed by incubation with primary antibodies antiCD86 (Solarbio, K010082P, 1:200) and anti- CD206 (Proteintech, 18704-1-AP, 1:200), respectively, at 4 °C overnight. The cells were then incubated with CoraLite488-conjugated secondary antibody (Proteintech, 1:200) and cy3-conjugated secondary antibody (Proteintech, 1:200), respectively, for 1 h in a humidified chamber at room temperature. The cell nuclei were counterstained with DAPI for 7 min. Images were obtained by the confocal laser scanning microscope and analyzed by ImageJ1.53.

#### 5.9.5 RNA sequencing (RNA-seq) and analysis

The cells were collected and total RNAs were extracted after induced by LPS for 48 h. RNA sequencing was performed by Novogene Co., Ltd (Beijing, China). Quality control checks were performed to confirm sequencing saturation and gene mapping distribution. Fragments per Kilobase of transcript per Million mapped reads (FPKM) value were used to express relative gene abundance. The stability and sensitivity of the genes were examined by evaluating the consistency of expression in the distinct groups. Read count data were standardized, and the significance and fold-change (p < 0.05 and log2 fold change > |1|) were set. The differences in expression were analyzed by DESeq2 R package (1.20.0). Cluster analysis was conducted using the clusterProfiler R package. Gene expression of standardized logFC was introduced to show the trends in different experimental conditions. DEGs were subjected to GO/KEGG functional pathway analyses after setting the significance threshold (p < 0.05). The genes having special trends were used to develop a network structure and were graphically represented using Cytoscape software with ClueGO + CluePedia plugin.

#### 5.9.6 Transmission electron microscopy (TEM)

Macrophages were co-cultured with magnesium particles in 24-well plates. Following a –4h incubation period, the cells were transferred to the optical microscope for observation, with images captured at 10-min intervals to document the phagocytosis process. Following phagocytosis of Mg particles by macrophages, the oil microscope images, TRITC-phalloidin staining images and DAPI staining images were recorded and superimposed. The oil microscope images were subsequently altered in color using Photoshop. The collected cells were fixed, embedded in resin, sectioned and examined under a TEM (Talos F200X, FEI Co., NLD).

### 5.10 Animal test procedures

#### 5.10.1 Surgical procedures

All animal procedures were conducted in accordance with the Laboratory Animal Ethics Guidelines of Huazhong University of Science and Technology (HUST). The experimental protocol was approved by the Institutional Animal Care and Use Committee (IACUC) of Tongji Medical College, HUST (IACUC No. 2605). Male Sprague-Dawley rats (100–150 g, n = 5) underwent bilateral extraction of the maxillary first molars under anesthesia. A bone defect (1.5 mm in diameter) was drilled at each extraction site using a dental implant handpiece. On one side, 1 mg of magnesium particles was implanted into the defect (Mg group), while the contralateral side received no treatment (control group). Following surgery, the animals were allowed free access to food and water. Maxillary bone specimens were harvested at 0 days, 1 week, 2 weeks, and 4 weeks post-surgery for subsequent analyses. Twelve healthy adult New Zealand White rabbits (2.5–3.0 kg, Hubei Yizhicheng Biotechnology CO., LTD.) were used to evaluate osseointegration in vivo. General anesthesia was induced with 40 mg/kg pentobarbital sodium via intravenous injection into the ear vein. The surgical sites were shaved, depilated, and disinfected with iodine. Under sterile conditions, a skin incision was made to expose the distal femur. A cylindrical implant bed (3 mm in diameter, 8 mm in depth) was prepared in the cortical bone using a dental drill under continuous saline irrigation. A titanium implant was then inserted into the defect. The surgical site was closed in layers (muscular fascia, subcutaneous tissue, and skin) using absorbable sutures. Postoperative antibiotic prophylaxis was administered via intramuscular injection of gentamicin (1 mL/day) for three consecutive days. The rabbits were monitored for eight weeks, during which they had ad libitum access to food and water. At the endpoint, the rabbits were euthanized by intravenous air embolism, and femoral specimens were harvested for further analysis.

#### 5.10.2 Micro-CT analysis

Specimens were fixed in PFA (4%) and subsequently transferred to ethanol (75%) for micro-computed tomography (micro-CT) scanning using a SkyScan 1176 system (Bruker, Germany) at a voxel resolution of 9 μm. Tomographic images were reconstructed using NRecon software. The bone defect region in the maxillary bone was visualized and assessed with DataViewer software, and quantitative analysis was performed using CTAn software. The following morphometric parameters were measured within the region of interest (ROI):Bone volume fraction (BV/TV), Trabecular thickness (Tb.Th), Trabecular number (Tb.N), and Trabecular separation (Tb.Sp). For evaluation of peri-implant biological tissue in the rabbit model, VGStudio Max 2.1 (Volume Graphics, Germany) was used to reconstruct three-dimensional images of the tissue surrounding the implant. The ROI was defined as a cylinder with dimensions Φ3.5 × L8.5 mm^3^ minus the implant volume (Φ3 × L8.5 mm^3^). Within this ROI, the following parameters were quantified: Biological tissue volume to total volume (BTV/TV), Biological tissue surface area to biological tissue volume (BTSA/BTV). These analyses enabled a comprehensive evaluation of both defect regeneration and peri-implant osseointegration.

#### 5.10.3 Histological and immunohistochemical analysis

Following fixation in paraformaldehyde (4%), the specimens were decalcified in ethylenediaminetetraacetic acid (EDTA, 10%), dehydrated through a graded ethanol series, embedded in paraffin, and coronally sectioned at a thickness of 4 μm. Tissue sections were stained with hematoxylin and eosin (H&E) and Masson's trichrome to evaluate overall tissue morphology and collagen deposition, respectively. For assessment of osteogenic activity, immunohistochemical staining was performed to detect collagen type I (COL-1) and osteopontin (OPN) at 2 and 4 weeks post-surgery. To evaluate the local inflammatory status, immunofluorescence staining for inducible nitric oxide synthase (iNOS) and arginase-1 (ARG1) was conducted at 1 week. Fluorescence images were acquired using a Nikon A1 confocal laser scanning microscope and analyzed using ImageJ software. The tibias containing the implants were dehydrated in a graded series of ethanol concentrations and subsequently embedded in methylmethacrylate resin (Technovit 7200 VLC, Exakt, Germany). A cutting and grinding unit (Exakt Apparatebau, Norderstedt, Germany) was employed to cut and grind the blocks, commencing from the major axis, until a final thickness of approximately 50 μm was reached. Transverse tissue sections were subjected to staining with methylene blue-acid fuchsin and alizarin red, in order to facilitate histological analysis of the bone tissue. A histological examination was conducted using the optical microscope with a standard light source. The bone-implant contact rate (BIC) was employed as a measure of the bone tissue surrounding the implant. The following markers were employed to quantify the bone tissue surrounding the implant in the ROI, which encompassed a complete four-section thread in contact with the bone at the tip of the implant, measuring 200 μm in width: BIC of ROI, bone area (BA), and bone mineralized area (BMA). ImageJ 1.53 software was utilized for image analysis.

### 5.11 Statistical analysis

Differences between two groups were assessed using two-tailed t-tests. The effects of different treatments, time points, and their interactions were assessed by using two-way analysis of variance (ANOVA), unless stated otherwise. GraphPad Prism 8 (Prism) was used for statistical analyses. Variability in plots and graphs is presented as SEM. All p < 0.05 were considered to be significant. ∗p < 0.05; ∗∗p < 0.01; ∗∗∗p < 0.001; ∗∗∗∗p < 0.0001.

## CRediT authorship contribution statement

**Zhixiang Nie:** Data curation, Investigation, Methodology, Writing – original draft. **Yasheng Sun:** Data curation, Investigation, Methodology, Writing – original draft. **Ke Li:** Data curation, Formal analysis, Investigation, Methodology. **Jukka P. Matinlinna:** Conceptualization, Formal analysis, Methodology, Project administration, Supervision, Writing – review & editing. **William M. Palin:** Formal analysis, Validation, Writing – review & editing. **Liam M. Grover:** Conceptualization, Funding acquisition, Supervision, Writing – review & editing. **Zhen Zhang:** Conceptualization, Formal analysis, Funding acquisition, Investigation, Methodology, Project administration, Resources, Supervision, Validation, Writing – original draft, Writing – review & editing.

## Declaration of competing interest

Authors declare that they have no competing interests.

## Data Availability

Data will be made available on request.
