# Supplementary material for: Non-destructive debridement and tuneable ion release via magnesium abrasion and electro-dissolution promote bone regeneration and osseointegration of infected implants
Source: Mater Today Bio. 2026 Apr 30;38:103183. doi: 10.1016/j.mtbio.2026.103183 (PMC13196099; doi:10.1016/j.mtbio.2026.103183)
Supplement: Multimedia component 1 [file mmc1.docx]

**Supporting information**

Non-destructive debridement and tuneable ion release via magnesium abrasion and electro-dissolution promote bone regeneration and osseointegration of infected implants

*Zhixiang Nie, Yasheng Sun, Ke Li, Jukka P. Matinlinna*, William M. Palin*, Liam M. Grover*, Zhen Zhang**

Z. Nie, Y. Sun, K. Li, Z. Zhang

Department of Stomatology, Union Hospital, Tongji Medical College, Huazhong University of Science and Technology, Wuhan 430022, China

Email: [zhangzhentitanium@163.com](mailto:zhangzhentitanium@163.com).

Y. Sun, K. Li, Z. Zhang

School of Stomatology, Tongji Medical College, Huazhong University of Science and Technology, Wuhan 430030, China

Email: [zhangzhentitanium@163.com](mailto:zhangzhentitanium@163.com).

Z. Nie, Y. Sun, K. Li, Z. Zhang

Hubei Province Key Laboratory of Oral and Maxillofacial Development and Regeneration, Wuhan 430022, China

Email: [zhangzhentitanium@163.com](mailto:zhangzhentitanium@163.com).

Z. Nie

State Key Laboratory of Materials Processing and Die & Mould Technology, School of Materials Science and Engineering, Huazhong University of Science and Technology, Wuhan 430074, China

J. P. Matinlinna

Biomaterials Science, Division of Dentistry, School of Medical Sciences, The University of Manchester, M13 9PL, United Kingdom

Email: [jpmat@hku.hk](mailto:jpmat@hku.hk).

J. P. Matinlinna

Dental Materials Science, Applied Oral Sciences, Faculty of Dentistry, The University of Hong Kong, Hong Kong SAR

Email: [jpmat@hku.hk](mailto:jpmat@hku.hk).

W. M. Palin

University of Birmingham, College of Medical and Dental Sciences, Institute of Clinical Sciences, 5 Mill Pool Way, Edgbaston, Birmingham B5 7EG, UK

Email: [w.m.palin@bham.ac.uk](mailto:w.m.palin@bham.ac.uk).

L. M. Grover

Healthcare Technologies Institute, School of Chemical Engineering, University of Birmingham, Birmingham, UK

Email: [l.m.grover@bham.ac.uk](mailto:l.m.grover@bham.ac.uk).


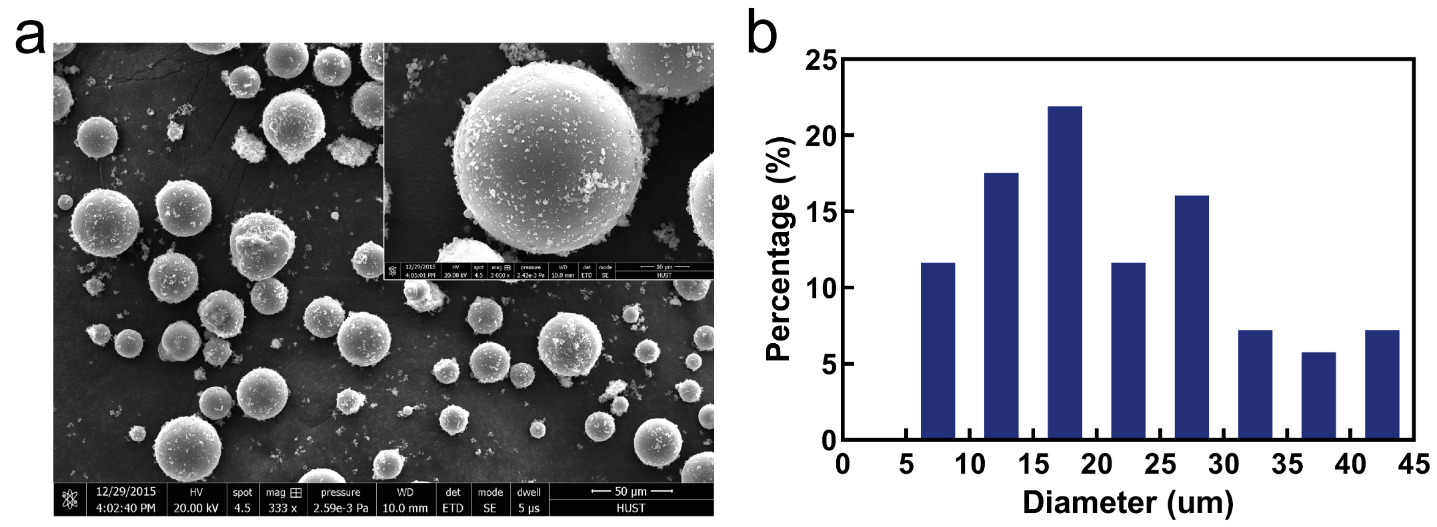


**Figure S1.** Spherical magnesium (Mg) powder particles used in MA and MAE. **a** SEM image of Mg powder particles. **b** Particle size analysis of Mg powder particles.


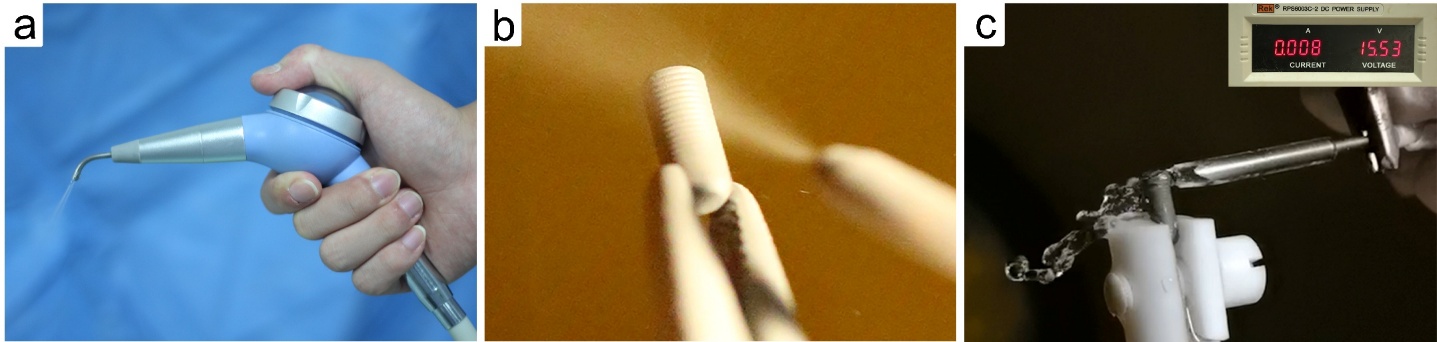


**Figure S2.** Photographs of the MA and MAE procedures. **a** The air-powered spray gun used for MA. **b** An implant undergoing MA treatment. **c** Residual magnesium particles on the implant surface were partially dissolved using charged normal saline during the electro-dissolution step of the MAE process.


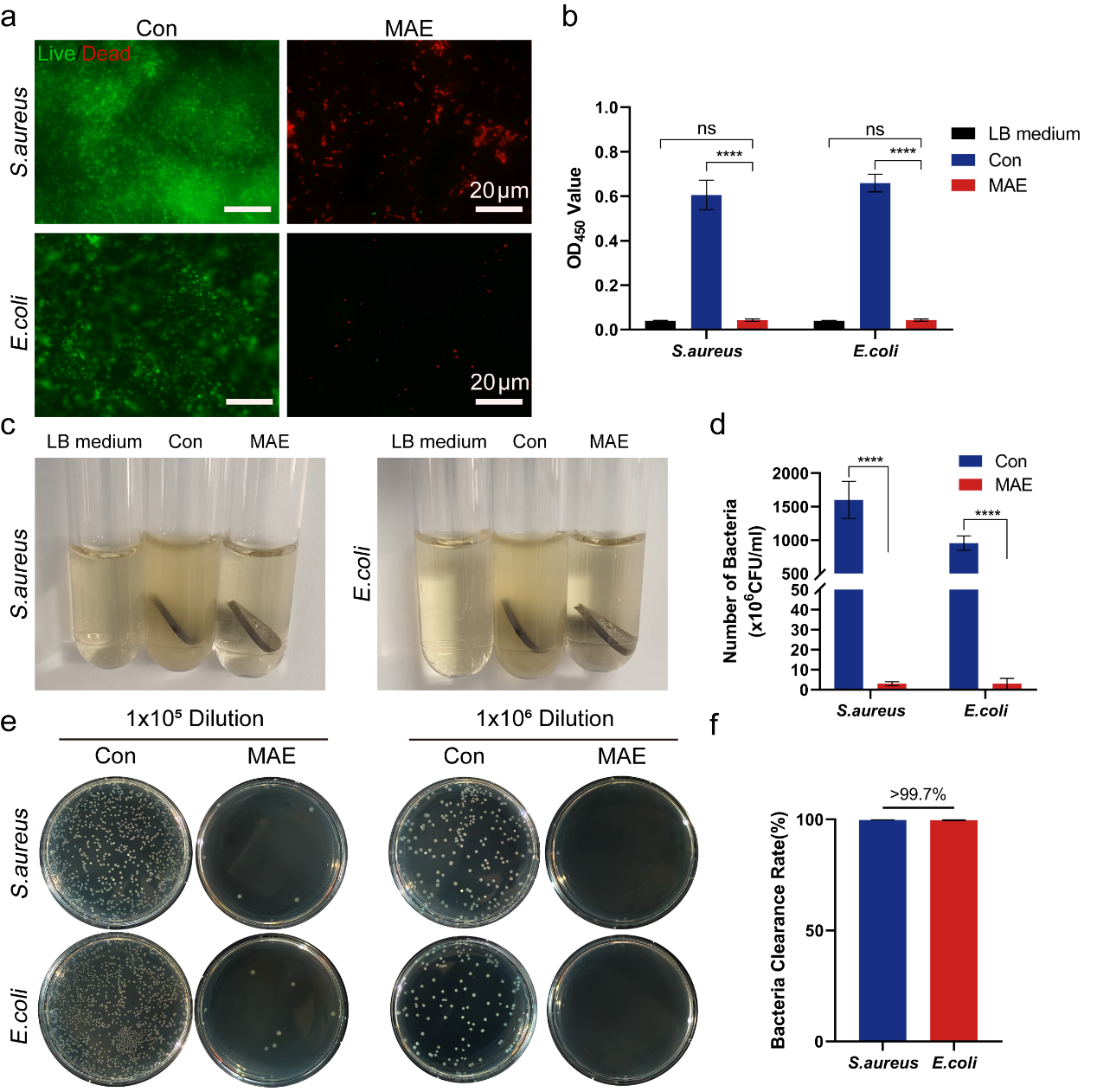


**Figure S3** MAE exhibits broad-spectrum antibacterial efficacy against Gram-positive and Gram-negative bacteria**. a** Representative live/dead fluorescence staining images of *S. aureus* and *E. coli* on implant surfaces before (Con) and after MAE treatment. Live (green), Dead (red) **b** Quantitative analysis of bacterial viability based on OD_450_ measurements after 12 h incubation following MAE treatment. **c** Representative photographs showing bacterial turbidity of *S. aureus* and *E. coli* cultured in LB medium after MAE treatment for 12 h. **d** Quantification of bacterial colony-forming units (CFU) for *S. aureus* and *E. coli* after MAE treatment. **e** Representative agar plate images of *S. aureus* and *E. coli* colonies at dilutions of 1×10⁵ and 1×10^6^ following MAE treatment. **f** Calculated bacterial clearance rates of *S. aureus* and *E. coli* based on CFU counts. Data are presented as mean ± SD (n = 3). ****P < 0.0001; ns, not significant.


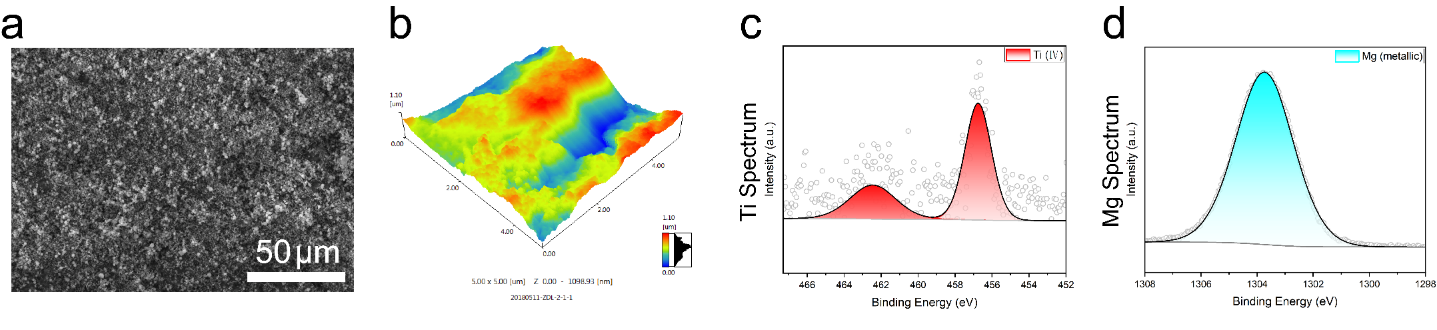
**Figure S4 Physical and chemical characterization of the sample surface after MA.** **a** SEM image of the sample surface in the MA group. **b** AFM analysis showing surface roughness of the MA-treated sample. **c, d** XPS analysis of the surface chemical composition after MA treatment.


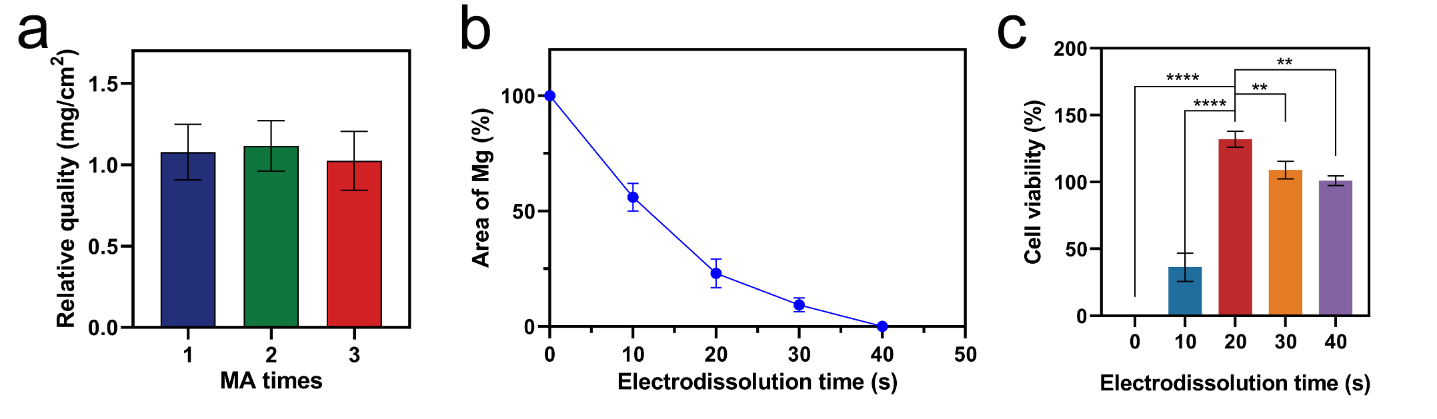


**Figure S5.** Surface properties after MA and MAE treatments. **a** The content of Mg is attached to the implant surface after repeated application. **b** The area of Mg particles retained on the surface after electrodissolution at different times. **c** Cell activity of BMSCs cultured on the MAE-treated surface for 7 days after electrodissolution at different times. These results indicated that some Mg particles were attached to the implant surface after MA treatment. After electrodissolution for 20 s, the Mg particles covered 20% of the surface, and the cells showed better activity. *n* = 3 per group. ***P* < 0.01, ****P* < 0.001, *****P* < 0.0001 by Student’s *t*-test. error bars = SD; data are presented as mean values ± SD


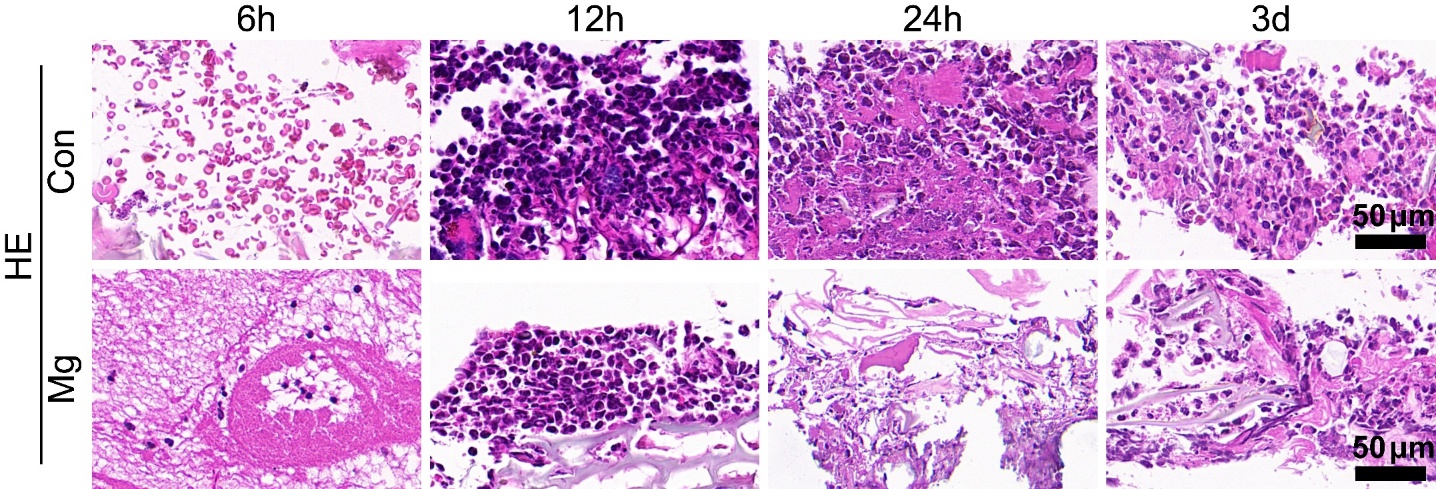


**Figure S6** H&E staining of the blood clot in the defect area was performed at 6 h, 12 h, 24 h, and 3 days after extraction of the maxillary first molar in rats.


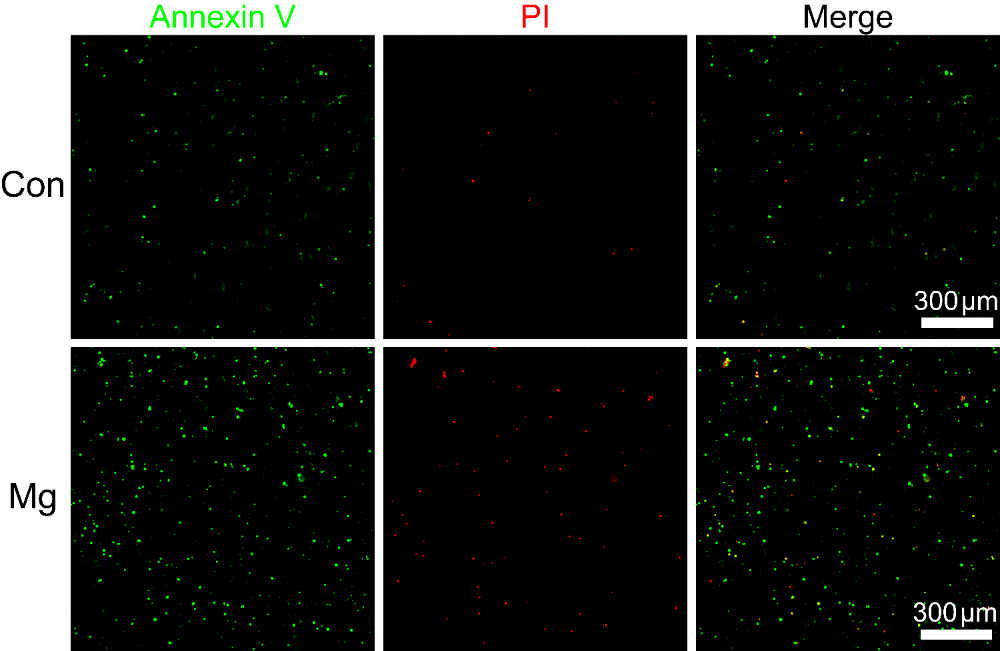


**Figure S7** Immunofluorescence images of neutrophil apoptosis after 4 hours of in vitro incubation with MAE-equivalent magnesium particles. Annexin V (Green), PI (Red).


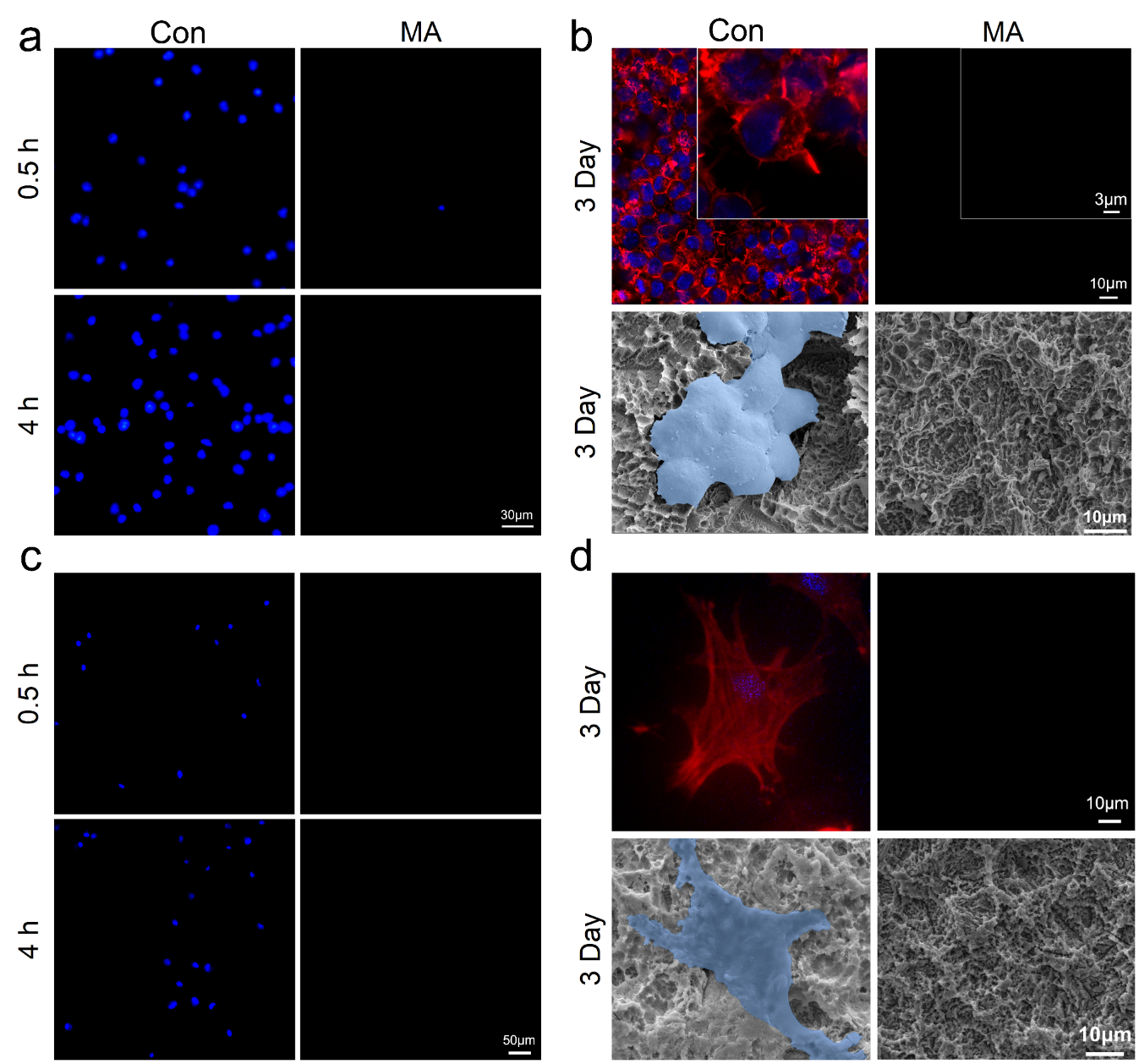


**Figure S8.** The influence of MA treatment on RAW264.7 and bone marrow mesenchymal stem cells. **a** Macrophage adhesion experiments were performed on the material surface for 0.5 h and 4 h. **b** Cell morphology of Macrophages on the surface after 3 days. **c** BMSCs adhesion experiments were performed on the material surface for 0.5 h and 4 h. **d** Cell morphology of BMSC on the surface after 3 days.


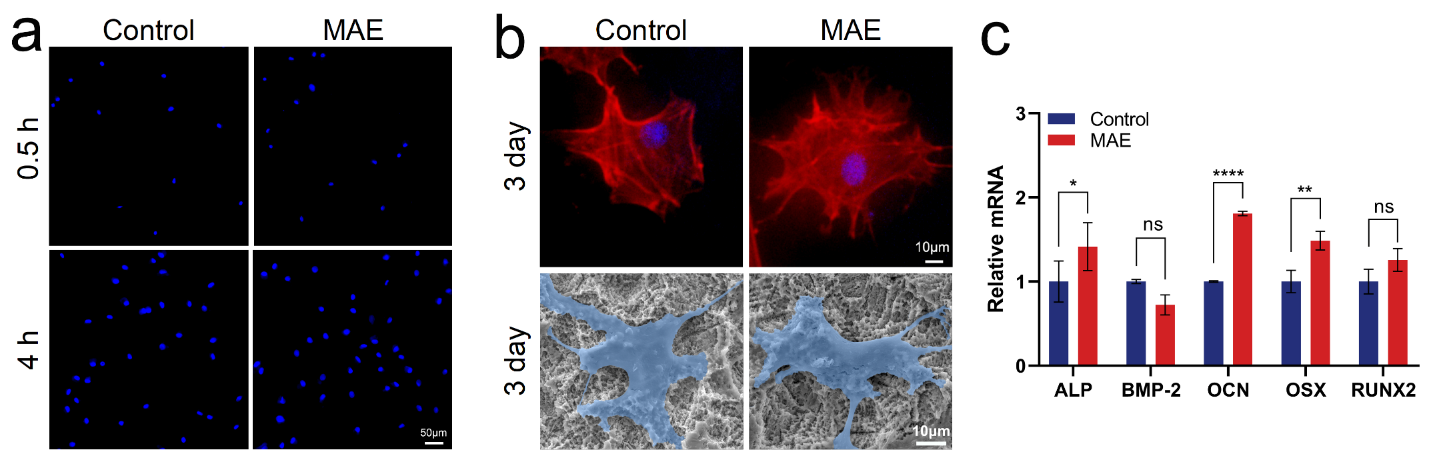


**Figure S9.** Behavior of BMSCs on the MAE-treated surface. **a** Adhesion of BMSCs on the material surface at 0.5 h and 4 h post-seeding. **b** Cell morphology of BMSCs on the surface after 3 days of culture. **c** Effects of surface physicochemical properties on the expression of osteogenic genes in BMSCs. *n* = 3 per group. *ns*, not significant, **P*<0.05, ***P* < 0.01, ****P* < 0.001 by Student’s *t*-test. error bars = SD; data are presented as mean values ± SD


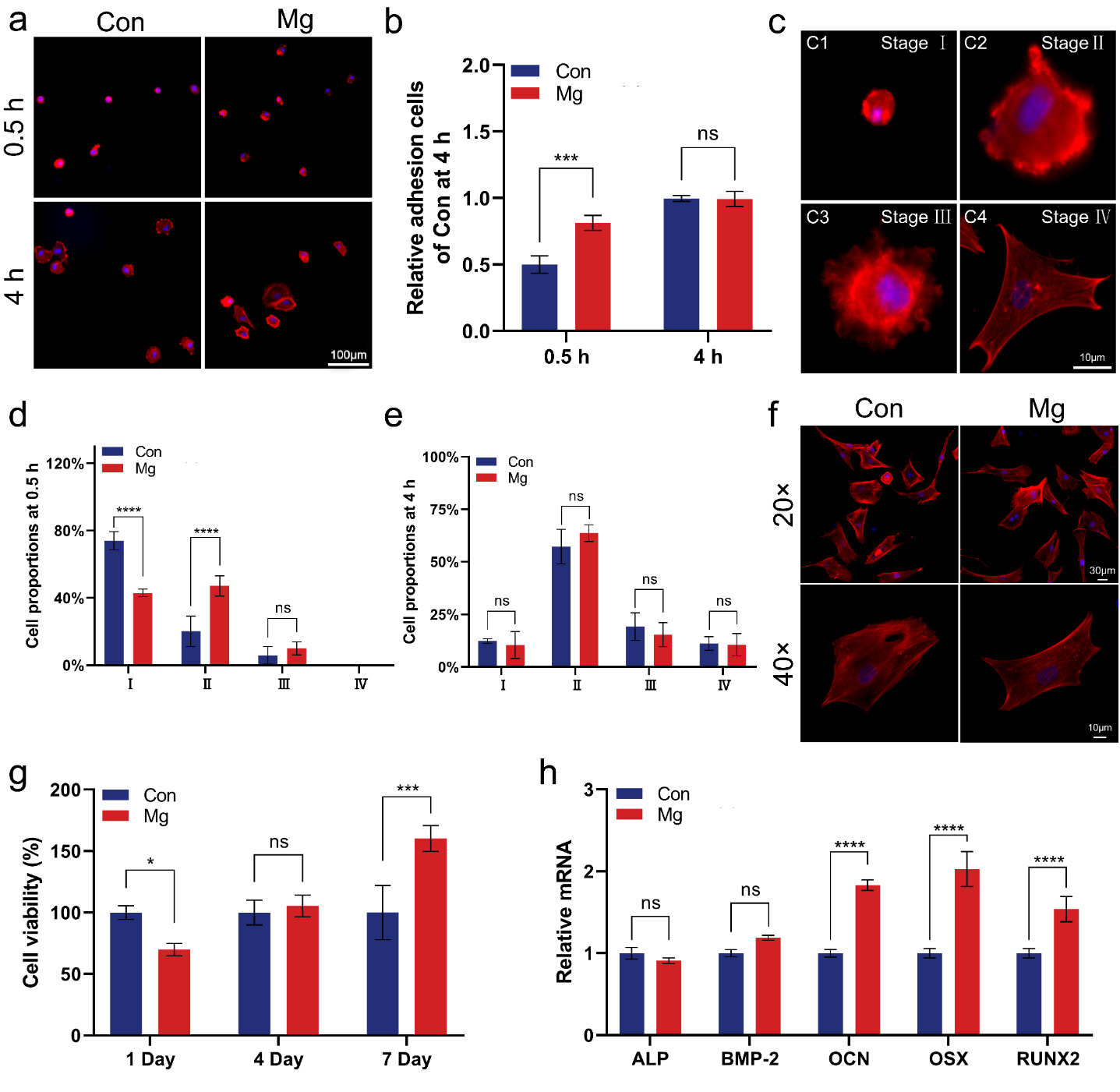


**Figure S10. The influence of Mg particles on BMSCs activity and function.** **a** Fluorescence images showing cell adhesion at 0.5 h and 4 h post-seeding. F-actin (red), nuclei (blue). **b** Quantification of adherent cell numbers. **c** Representative fluorescence images of cell adhesion morphology at different stages: **c1** round cells (Stage I), **c2** round cells with filopodia (Stage II), **c3** cells with cytoplasmic webbing (Stage III), **c4** well-spread cells (Stage IV). **d, e** Proportions of cells at different adhesion stages after 0.5 h and 4 h. **f** Cytoskeletal organization of BMSCs cultured with Mg particles after 24 h. **g** Quantification of adherent cells after 24 h. **h** Relative expression of osteogenic genes in BMSCs cultured with Mg particles. n = 3 per group. Data are presented as mean ± SD. **P* < 0.05, ***P* < 0.01, ***P < 0.001, *****P* < 0.0001 by student’s *t*-test; ns, not significant.


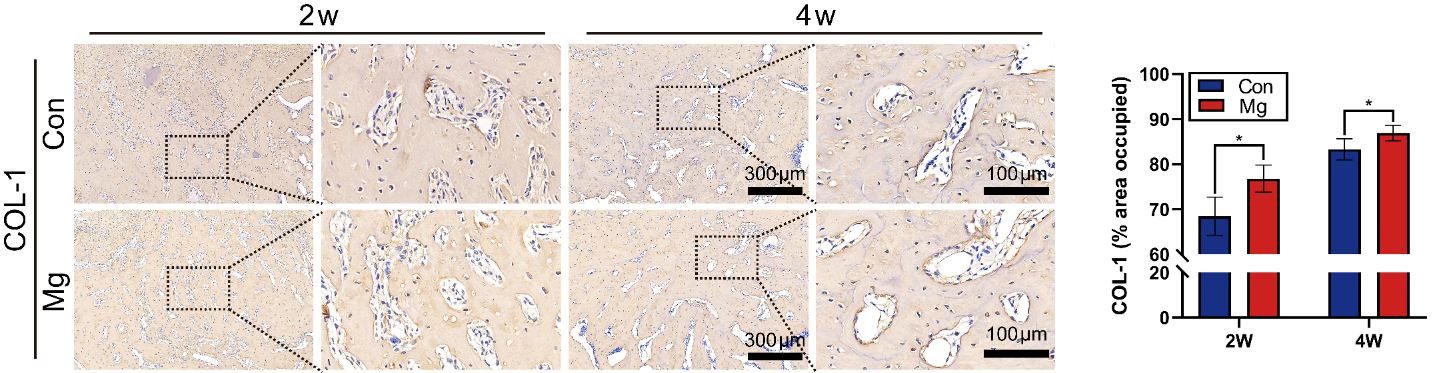


**Figure S11**. Representative immunohistochemical staining of collagen type I (COL-1) and corresponding semi-quantitative analysis (n = 4); boxed regions are enlarged on the right. Data are presented as mean ± SD. **P* ≤ 0.05 by student’s *t*-test.


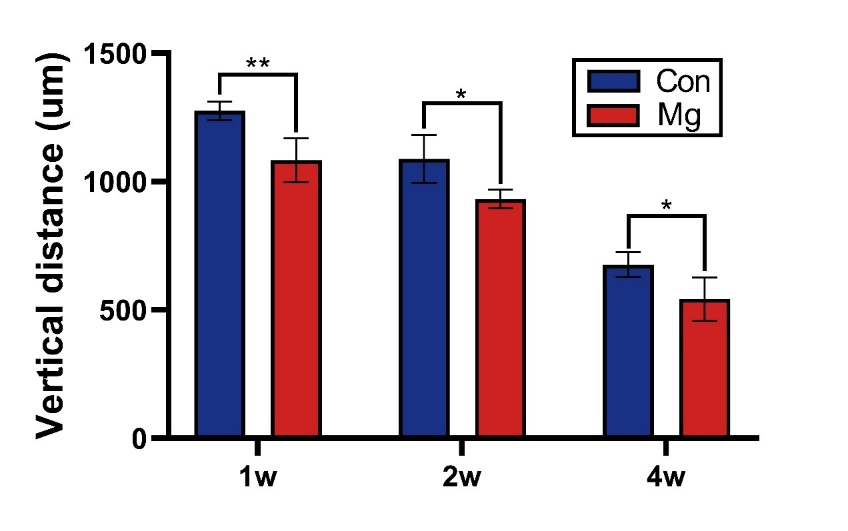


**Figure S12** Quantification of the vertical distance from the nadir of the osseous defect to the stratum spinosum.


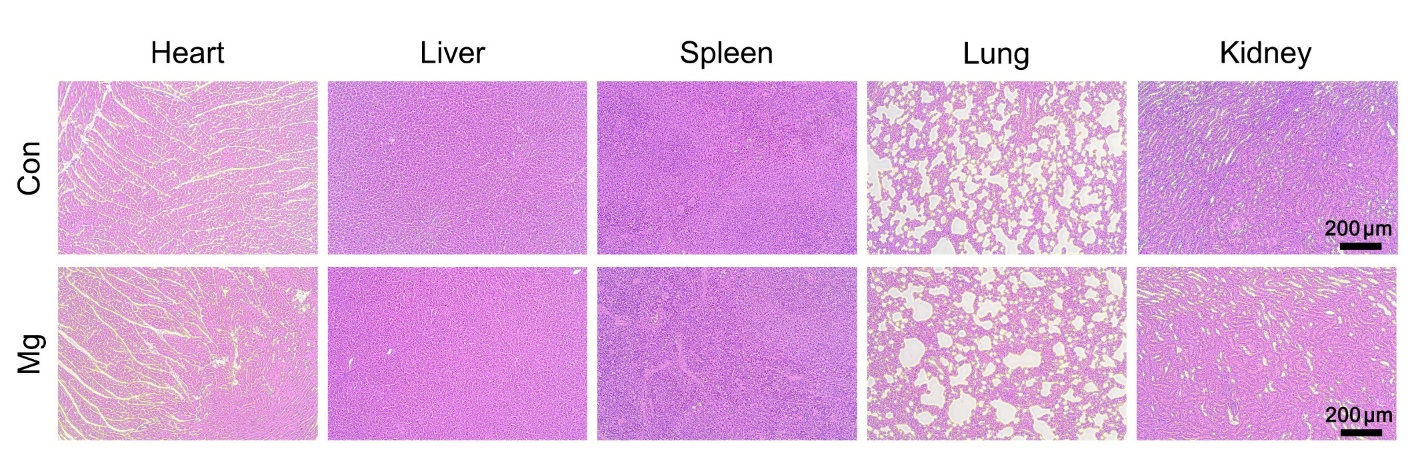


**Figure S13.** The histopathologic difference in normal organ sections was investigated using H&E staining method. Representative H&E staining of the heart, liver, spleen, lungs, and kidneys at 4 weeks post-implantation revealed no apparent solid lesions or histopathological abnormalities in either group, indicating favorable biocompatibility and systemic safety of the implanted magnesium particles.

**Table S1.** Comparison of representative treatment options for oral peri-implantitis.

| Treatment of peri-implantitis | Titanium curette | Titanium brusher | Glycine air-powder | Antimicrobial | Er: YAG laser | **MAE** |
| --- | --- | --- | --- | --- | --- | --- |
| Absence of surface damage | × | × | √ | √ | √ | **√** |
| Bacteria removal | × | √ | √ | × | × | **√** |
| In favor of osteogenesis | × | × | × | × | × | **√** |

**Table S2.** Primer sequences for qRT-PCR

| Primer | Forward (5'-3') | | Reverse (5'-3') |
| --- | --- | --- | --- |
| M-CCL2 | | GTGCTGACCCCAAGAAGGAAT | TTGAGGTGGTTGTGGAAAAGG |
| M-CD86 | | GGGCTCGTATGATTGTTT | CTTCTTAGGTTTCGGGTG |
| M-IL-1β | | AGCATCCAGCTTCAAATC | CTTCTCCACAGCCACAAT |
| M-IL-6 | | TTGCCTTCTTGGGACTGAT | TTGCCATTGCACAACTCTT |
| M-CD206 | | ACCCAAGGGCTCTTCTAA | TGGCCTCTTGAGGTATGT |
| M-IL-4 | | GAGACTCTTTCGGGCTTTT | TGCTCTTTAGGCTTTCCAG |
| M-IL-10 | | TTTCAAACAAAGGACCAG | GGATCATTTCCGATAAGG |
| M-TGF-β | | TCACTAGATCGCCCTTTC | ACAGCAACTTCTTCTCCC |
| R-ALP | | CGGGAAGCAAGGCAGAAGC | GCGGGGACCATAAGCGAGT |
| R-BMP-2 | | AGAAAAGCGTCAAGCCAA | CAGTCATTCCACCCCACA |
| R-OCN | | GAATAGACTCCGGCGCTACC | TCCTGGAAGCCAATGTGGTC |
| R-OSX | | CTCCTTGGTGGGACATGC | GTAGGCAGCTGGGGGTTC |
| R-RUNX2 | | GCGGTGCAAACTTTCTCCAG | TCACTGCACTGAAGAGGCTG |

**Table S3.** **Information of primary antibodies**

| Primary Antibody | Company | Cat No. |
| --- | --- | --- |
| MPO | Abcam | ab208670 |
| COL1 | Abcam | ab270993 |
| CD68 | Boster | BA3638 |
| ARG1 | Proteintech | 16001-1-AP |
| iNOS | Proteintech | 18985-1-AP |
| OPN | Servicebio | China |
| BAX | ABclonal | A19684 |
| Cleaved caspase-3 | ABclonal | A19664 |
| Caspase-3 | ABclonal | A11319 |
| Cleaved caspase-1 | ABclonal | A18646 |
| Caspase-1 | ABclonal | A16792 |
| MLKL | ABclonal | A5579 |
| Phospho-MLKL | ABclonal | AP1174 |
